# Langerin^+^ DCs regulate innate IL-17 production in the oral mucosa during *Candida albicans*-mediated infection

**DOI:** 10.1371/journal.ppat.1007069

**Published:** 2018-05-21

**Authors:** Florian Sparber, Tamas Dolowschiak, Sarah Mertens, Laura Lauener, Björn E. Clausen, Nicole Joller, Patrizia Stoitzner, Roxane Tussiwand, Salomé LeibundGut-Landmann

**Affiliations:** 1 Section of Immunology, Vetsuisse Faculty, University of Zürich, Zürich, Switzerland; 2 Institute of Experimental Immunology, University of Zürich, Zürich, Switzerland; 3 Institute for Molecular Medicine, University Medical Center of the Johannes Gutenberg-University Mainz, Mainz, Germany; 4 Department of Dermatology, Venereology & Allergology, Medical University Innsbruck, Innsbruck, Austria; 5 Department of Biomedicine, University of Basel, Basel, Switzerland; University of Wisconsin-Madison, UNITED STATES

## Abstract

The opportunistic fungal pathogen *Candida albicans* frequently causes diseases such as o*ropharyngeal candidiasis* (OPC) in immunocompromised individuals. Although it is well appreciated that the cytokine IL-17 is crucial for protective immunity against OPC, the cellular source and the regulation of this cytokine during infection are still a matter of debate. Here, we directly visualized IL-17 production in the tongue of experimentally infected mice, thereby demonstrating that this key cytokine is expressed by three complementary subsets of CD90^+^ leukocytes: RAG-dependent αβ and γδ T cells, as well as RAG-independent ILCs. To determine the regulation of IL-17 production at the onset of OPC, we investigated in detail the myeloid compartment of the tongue and found a heterogeneous and dynamic mononuclear phagocyte (MNP) network in the infected tongue that consists of Zbtb46^-^Langerin^-^ macrophages, Zbtb46^+^Langerin^+^ dendritic cells (DCs) and Ly6C^+^ inflammatory monocytes. Of those, the Langerin^+^ DC population stands out by its unique capacity to co-produce the cytokines IL-1β, IL-6 and IL-23, all of which promote IL-17 induction in response to *C*. *albicans* in the oral mucosa. The critical role of Langerin^+^ DCs for the innate IL-17 response was confirmed by depletion of this cellular subset *in vivo*, which compromised IL-17 induction during OPC. In conclusion, our work revealed key regulatory factors and their cellular sources of innate IL-17-dependent antifungal immunity in the oral mucosa.

## Author summary

IL-17 is a key cytokine for immune homeostasis and host defense in barrier tissues, which can also drive inflammatory diseases and immunopathology under certain conditions. Most studies addressing IL-17-mediated processes focus on the lower gastrointestinal tract, while other barrier tissues such as the oral mucosa remain largely understudied, despite their important role for entry of hazardous microbes. The protective role of IL-17 is particularly relevant for host defense against the fungal pathogen *Candida albicans*, as evidenced by individuals with genetic defects in the IL-17 pathway. Experiments with mice demonstrated that rapid IL-17 expression is essential for fungal control during oropharyngeal candidiasis. How this is regulated remained largely unclear. In this study, we identified a tripartite population of innate lymphocytic cells as the *bona fide* cellular source of IL-17 in the oral cavity. At the molecular level, we found IL-6 and IL-1 to act synergistically and complementary to IL-23 for instruction of IL-17 in response to infection. In search for the cellular source(s) of these IL-17-inducing factors we identified Langerin^+^ DCs as key players in the coordination of *C*. *albicans*-induced innate IL-17 production. Together, this greatly advances our understanding of IL-17 regulatory mechanisms in antifungal immunity in the oral mucosa.

## Introduction

As part of the upper gastrointestinal tract, the oral cavity is colonized by microbes and constitutes an important entry point for hazardous pathogens. However, despite the relevance of the oral mucosa as a first site of interaction between microbes and the host, it remains little studied and its cellular composition is not well characterized. *Oropharyngeal candidiasis* (OPC) is a common infection of the oral cavity mediated by the opportunistic fungal pathogen *Candida albicans* in immunocompromised individuals [1]. It frequently develops as a consequence of impaired immune function due to administration of steroids and other immunosuppressant agents, or because of underlying diseases such as AIDS or primary immunodeficiencies [1]. The recent study of hereditary factors predisposing to OPC and other forms of mucocutaneous candidiasis determined the relevance of the interleukin-17 (IL-17) pathway as a key mechanism for protective immunity against this disease. Genes directly associated with disease include those encoding the IL-17 receptor subunits IL-17RA [2] and IL-17RC [3], the signaling adaptor Act1 (also referred to as CIKS or TRAF3IP2) [4] and the cytokine family member IL-17F [2], but also genes encoding transcription factors involved in the regulation of IL-17 production, such as STAT1 [5,6], STAT3 [7–9] and RORγt [10]. Work on experimental mouse models further confirmed the important role of IL-17, in particular IL-17A and IL-17F, in antifungal defense [11–13].

IL-17 is thought to act by promoting the antimicrobial function and the epithelial integrity of barrier tissues [14,15]. Although there is little doubt about the relevance of IL-17 in host-defense against *C*. *albicans*, the tissue-specific regulation of IL-17 production during candidiasis remains not well understood. Th17 cells are the major source of IL-17A and IL-17F in response to *C*. *albicans* [16–19]. The rapid induction of IL-17A and IL-17F within 24 hours post-infection in experimentally infected mice suggested that innate cells also participate in cytokine production in the infected mucosa. Indeed, previous work from our group demonstrated that *Rag1*^*-/*-^ animals, lacking T and B cells, control the fungus and recover from infection within a week [13]. Genetic deletion or antibody-mediated depletion of CD90^+^ or IL-2 receptor gamma chain-dependent cells in *Rag1*^*-/*-^ mice however rendered the animals susceptible to OPC to a degree similar to IL-17RA-deficient mice [13]. Hence, our oberservations strongly argued for the involvement of a RAG-independent cellular source of IL-17. This result was challenged by a study using an IL-17A reporter mouse strain suggesting that T cells produce IL-17 within 24 hours of primary infection [20]. Here we employed a flow cytometry approach to directly visualize IL-17A protein expression in the tongue of infected mice and identified three separate populations of IL-17-producing CD90^+^ leukocytes that act in an at least partially redundant manner during acute OPC. Moreover, we dissected and tested the functional relevance of different mononuclear phagocytes and IL-17 instructive cytokines in the infected tissue and thereby revealed the key determinants that orchestrate the innate IL-17 response against *C*. *albicans* in the oral mucosa.

## Results

### IL-17 is produced by a tripartite population of CD90^+^ leukocytes during acute OPC

**Fig 1:**
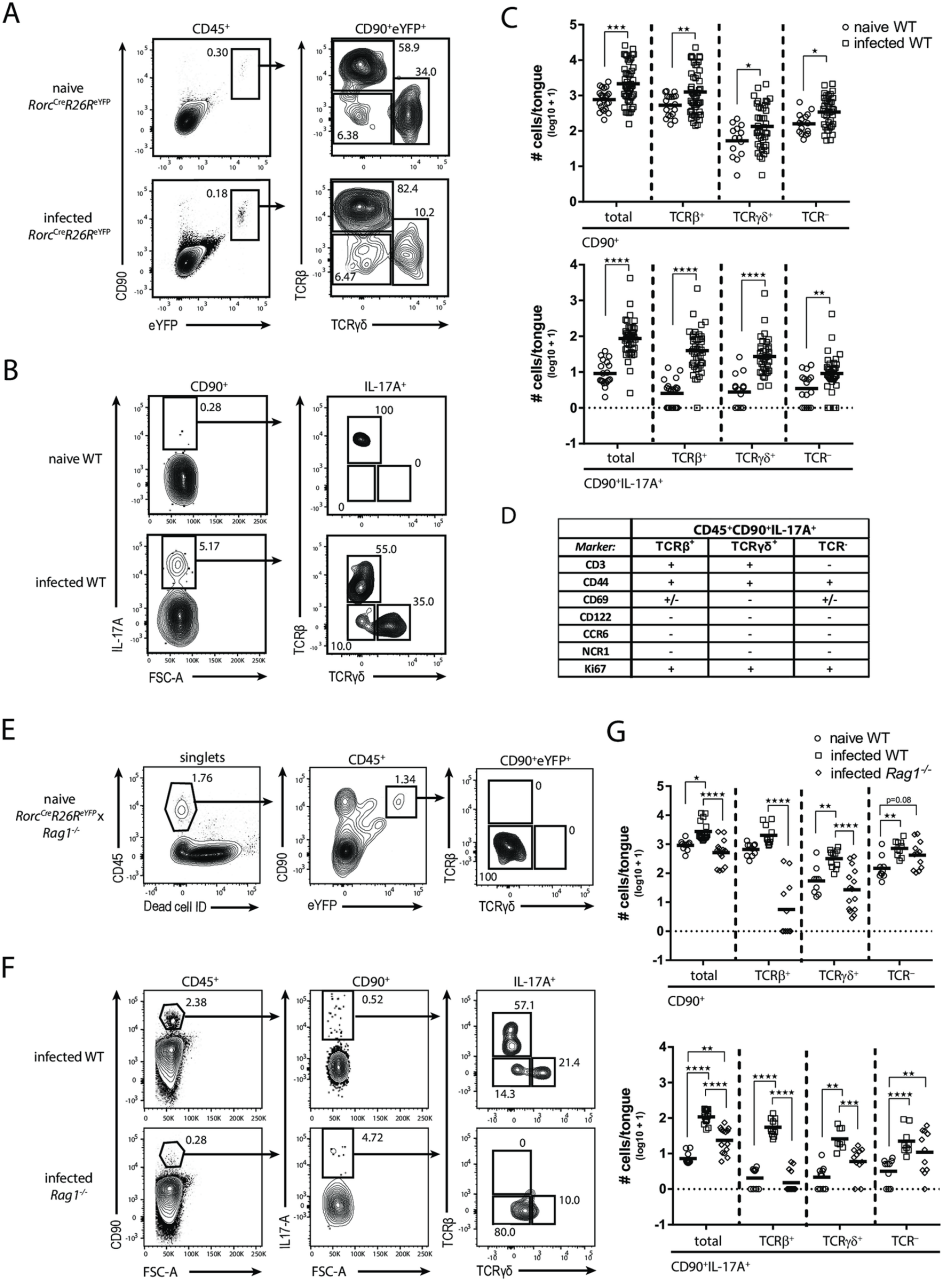
IL-17A is expressed by a tripartite population of CD90^+^ leukocytes during acute OPC. (**A**) CD90^+^eYFP^+^ cells in the tongue of naïve and infected *Rorc*^*Cre*^*R26R*^*eYFP*^ mice were analyzed for TCRβ and TCRγδ expression. Pre-gating is on CD45^+^ cells. Representative FACS plots from one out of two independent experiments are shown. Numbers indicate the % of parent in each gate. (**B**) Representative analysis of the CD90^+^IL-17A^+^ subsets in the tongue of naïve and infected WT animals. Pre-gating is on CD45^+^CD90^+^ cells. Numbers indicate the % of parent in each gate. (**C**) Summary plot with absolute numbers of CD90^+^ and CD90^+^IL-17A^+^ cells and the respective TCRβ^+^, TCRγδ^+^ and TCR^-^ subsets according to the analysis shown in **B**. Each symbol represents one animal, the mean of each group is indicated. Graphs show pooled data from 12 independent experiments. (**D**) Surface and proliferation markers expressed by the three CD90^+^IL-17A^+^ subsets in the infected tongue of WT mice. (**E**) Analysis of CD90^+^eYFP^+^ cells in the tongue of naïve *Rorc*^*Cre*^*R26R*^*eYFP*^ x *Rag1*^*-/-*^ mice. Pre-gating is on single and viable cells. Numbers indicate the % of parent in each gate. Representative plots from one out of two independent experiments are shown. (**F**) Representative analysis of CD90^+^IL-17A^+^ subsets in the tongue of infected WT and *Rag1*^*-/-*^ mice. Pre-gating is on CD45^+^ cells. Numbers indicate the % of parent in each gate. (**G**) Summary plots with absolute numbers of CD90^+^ and CD90^+^IL-17A^+^ cells and the respective TCRβ^+^, TCRγδ^+^ and TCR^-^ subsets according to the analysis shown in **F**. Each symbol represents one animal, the mean of each group is indicated. Graphs show pooled data from four independent experiments. *p<0.05; **p<0.01, ***p<0.001; ****p<0.0001. See also S1 and S2 Figs.

We set out to characterize cells with the potential to produce IL-17A and IL-17F in response to *C*. *albicans* at the site of infection. To do so, we analyzed tongue leukocytes from *Rorc*^*Cre*^*R26R*^*eYF*P^ reporter mice for the expression of eYFP as a reporter for the lymphocyte-associated transcription factor RORγt. We detected a distinct population of eYFP^+^ cells that uniformly co-expressed CD90^+^ in both naïve and infected tongues and comprised αβ T cells, γδ T cells and TCR^-^ ILCs (Fig 1A). Innate IL-17 production in the murine tongue was so far never demonstrated directly and at the single cell level. We therefore established a protocol to visualize IL-17A and IL-17F protein in the *C*. *albicans*-infected tongue by combining *in vivo* Brefeldin A administration and intracellular cytokine staining (S1A Fig). By doing so we detected a well-defined population of CD90^+^IL-17A^+^ cells in the tongue of infected but not naïve wild type (WT) mice (Fig 1B). Because *Il17a* and *Il17f* transcript levels are maximal on day 1 post-infection with *C*. *albicans* strain SC5314 [13], we focused our analysis on this time point. Reminiscent of our analysis of *Rorc*^*Cre*^*R26R*^*eYF*P^ mice, the CD90^+^IL-17A^+^ population comprised three subsets: αβ T cells, γδ T cells and TCR^-^ ILCs (Fig 1B). The specificity of our IL-17A staining in CD90^+^ cells was confirmed by applying the same experimental conditions to *Il17af*^*-/-*^ mice, which served as a biological negative control (S1B Fig). In WT mice, the majority of IL-17A^+^ cells co-produced IL-17F (S1C Fig). NKT cells were not found to contribute significantly to IL-17A production in the infected tongue, as only very few IL-17A^+^ cells could be stained with CD1d tetramers (S1D Fig). Quantification of the CD90^+^ and CD90^+^IL-17A^+^ subpopulations revealed an expansion of all subsets within the first 24 hours of infection (Fig 1C). Consistent with this, all IL-17A^+^ cells stained positive for the marker Ki67 (Fig 1D and S2 Fig), suggesting *in situ* proliferation of the three IL-17A-producing cellular subsets. To characterize the IL-17A-producing cells in the tongue of OPC-infected mice in more detail, we analyzed phenotypic and activation markers on their surface. Consistent with their TCR expression, αβ and γδ T cells, but not TCR^-^ ILCs, co-expressed CD3 (Fig 1D and S2 Fig). All three IL-17A^+^ subsets displayed an activated phenotype as evidenced by their high expression of CD44 and partial expression of CD69. None of the IL-17A-expressing cellular subsets expressed CD122, NCR1, CCR6 or MHCII (S2 Fig).

To further define the ILC compartment, we examined RAG1-deficient mice. *Rorc*^*Cre*^*R26R*^*eYFP*^ fate reporter mice, crossed to a RAG1-deficient background, harbored a clear population of eYFP^+^ cells in the naïve tongue that were uniformly CD90^+^ (Fig 1E). Infection of (reporter-less) *Rag1*^*-/-*^ mice revealed, as expected, an overall reduction of total CD90^+^ and CD90^+^IL-17A^+^ cells due to the absence of TCRβ^+^ and TCRγδ^+^ cells. Yet, the number of IL-17A-producing cells was still significantly increased in infected as compared to naïve WT mice, which was attributed to the ILC subset (Fig 1F and 1G). Overall, our data demonstrate that direct visualization of IL-17A protein production by intracellular staining *ex vivo* revealed the involvement of three distinct CD90^+^ leukocyte subsets during OPC.

### IL-17A production relies on IL-23, IL-6 and IL-1 signaling in a partially redundant manner

**Fig 2:**
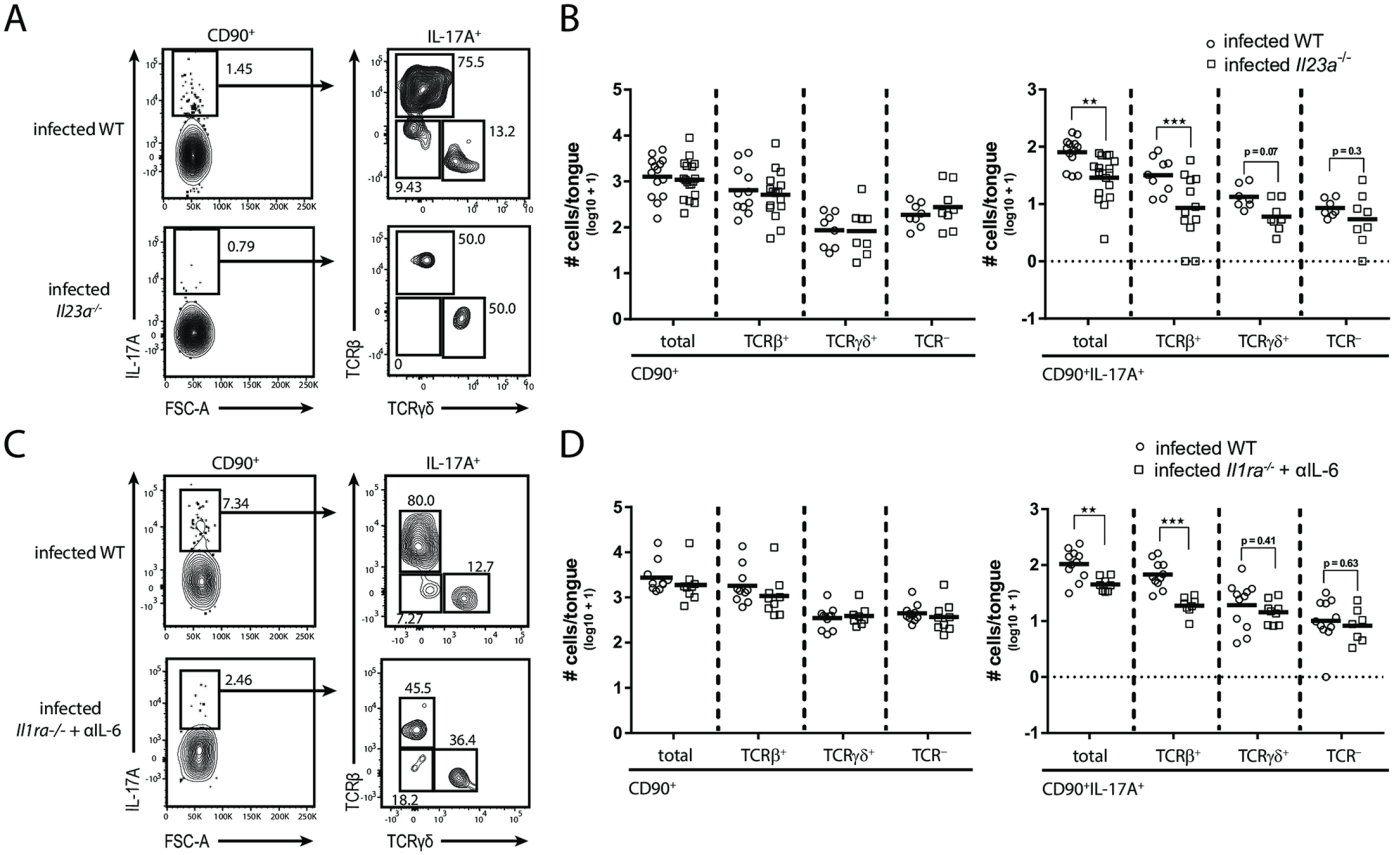
IL-17A production relies on IL-23, IL-6 and IL-1 in a partially redundant manner. IL-17A^+^ cellular subsets in the tongue of infected WT and *Il23a*^*-/-*^ animals (**A**-**B**) or infected *Il1ra*^*-/-*^ mice that were treated with anti-IL-6 antibody and WT controls (**C**-**D**). (**A**, **C**) Representative plots. Pre-gating is on CD45^+^CD90^+^ cells. Numbers indicate the % of parent in each gate. (**B**, **D**) Summary graphs with the total CD90^+^ and CD90^+^IL-17A^+^ cells as well as the respective TCRβ^+^, TCRγδ^+^ and TCR^-^ subsets according to the analysis shown in **A** and **C**. Each symbol represents one animal, the mean of each group is indicated. Graphs show pooled data from three (**D**) or four (**C**) independent experiments. (**p<0.01; ***p<0.001). See also S3 Fig.

IL-23 is a key regulator of IL-17 immunity. To investigate the impact of IL-23 on IL-17A production by the three distinct cellular subsets during OPC, we infected *Il23a*^*-/-*^ animals in comparison to WT controls and assessed the changes in absolute numbers of IL-17A producing CD90^+^ cells and the corresponding TCRβ^+^, TCRγδ^+^ and ILC subsets in the tongue on day 1 post infection. While the CD90^+^ populations overall remained unchanged in *Il23a*^*-/-*^ mice in comparison to WT controls, the number of IL-17A-positive cells was significantly reduced (Fig 2A and 2B). This is consistent with previous studies analyzing the dependence of overall IL-17A and IL-17F expression on IL-23 in the infected organ [13] and the impact of IL-23 on fungal control [11,13]. Of the three identfitied IL-17A-producing cellular subsets, the TCRβ^+^ subset was most strongly affected, but also the TCRγδ^+^ and ILC populations showed a clear trend towards reduced IL-17A production in IL23-deficient mice compared to WT controls.

Instruction of IL-17A production by the adaptive immune system is driven by IL-1β and IL-6 in addition to IL-23 [21–23]. The impact of these cytokines on the regulation of IL-17A production by innate immune cells during OPC was not investigated in detail so far. Therefore, we set out to analyze mice with a nonfunctional IL-1 and/or IL-6 pathway. While genetic deletion of the IL-1 receptor or antibody-mediated neutralization of IL-6 alone had no measurable effect on IL-17A induction (S3 Fig), the combination of both resulted in a drop in CD90^+^IL-17A^+^ cells in the tongue of infected mice in comparison to controls (Fig 2C and 2D). Again, the effect was most pronounced for the TCRβ^+^ subset. Together, these data demonstrate that IL-23, IL-1 and IL-6 play a critical role for rapid IL-17A induction at the onset of OPC, whereby IL-6 and IL-1 act in a redundant manner.

### The MNP network in the tongue undergoes dynamic changes during acute OPC

**Fig 3:**
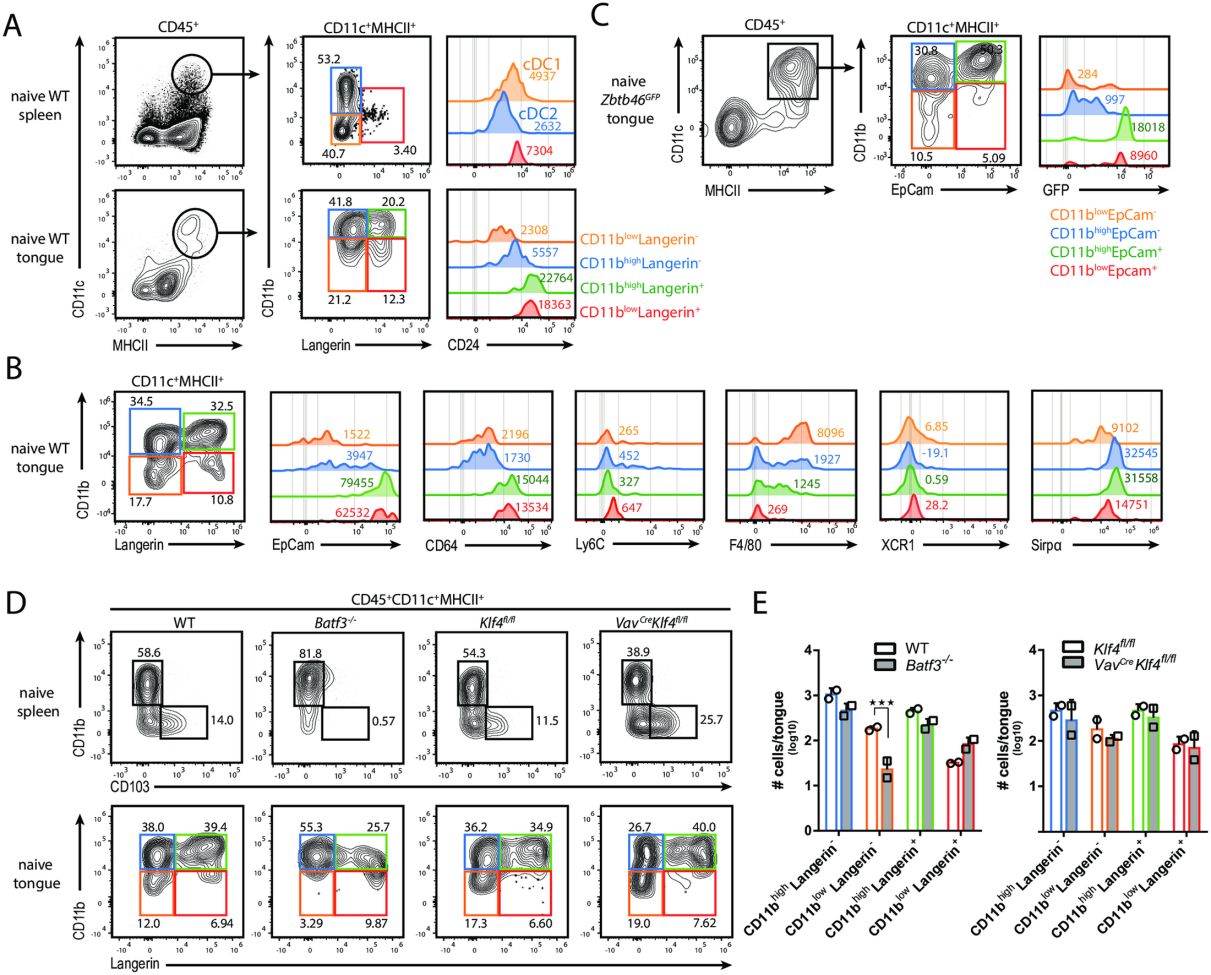
The MNP network in the tongue during steady state. (**A**) Phenotypic characterization of tongue-resident CD11c^+^MHCII^+^ MNPs in naïve WT mice. Spleen cells were also analyzed as a control. On the basis of CD11b and Langerin expression, CD11c^+^MHCII^+^ MNPs were divided in three (in the spleen, upper row) or four subsets (in the tongue, lower row), respectively, and analyzed for CD24 expression. (**B**) Tongue-resident CD11c^+^MHCII^+^ MNP subsets in naïve WT mice were analyzed for the expression of EpCam, CD64, Ly6C, F4/80, XCR1 and Sirpα. FACS plots are representative of one out of three independent experiments with 2 animals each. Pre-gating is on CD45^+^CD11c^+^MHCII^+^ MNPs. (**C**) Tongue-resident CD11c^+^MHCII^+^ MNP subsets in naïve *Zbtb46*^*GFP*^ reporter mice were analyzed for the expression of GFP. FACS plots in **A** and **C** are representative of one out of two independent experiments with one animal each. Pre-gating is on CD45^+^ cells. Numbers in the histograms in **A** to **C** indicate MFI (median) of the indicated markers. (**D-E**) Comparison of spleen and tongue-resident CD11c^+^MHCII^+^ MNP subsets in WT versus *Batf3*^*-/-*^ and *Klf4*^*fl/fl*^ versus *Vav*^*Cre*^*Klf4*^*fl/fl*^ mice, respectively. Pre-gating is on CD45^+^MHCII^+^CD11c^+^ cells. Representative FACS plots are shown in **D** and summary graphs with absolute numbers of CD11c^+^MHCII^+^ MNP subsets in the indicated genotypes are shown in **E**. Each symbol represents one animal, the mean of each group is indicated. Data are representative of one out of two independent experiments. (***p<0.001). See also S4 Fig.

Mononuclear phagocytes (MNPs) are a prominent source of IL-1β, IL-6 and/or IL-23 in diverse infectious settings and they may thus represent important players in the regulation of innate IL-17A production during acute OPC. However, the CD11c^+^MHCII^+^ MNP network in the tongue of mice remains poorly characterized. We thus set out to dissect this cellular compartment in detail. This required adaptation of our protocol for mouse tongue preparation to assure that the tissue-resident cells were liberated from the dense epithelial network. Based on CD11b and Langerin (CD207) expression, we identified four distinct subsets within the CD11c^+^MHCII^+^ population in the naïve tongue (Fig 3A). Unexpectedly, these cells appeared to be clearly distinct from the established DC populations e.g. in the spleen that comprise CD11b^-^CD24^+^ conventional DCs group 1 (cDC1s), CD11b^+^CD24^-^ cDC2s [24] and Langerin^+^ DCs [25] (Fig 3A). Further characterization of the CD11c^+^MHCII^+^ MNPs in the naïve tongue showed that all four MNP subsets expressed low levels of XCR1 and Ly6C and high levels of Sirpα, whereby the highest expression of Sirpα was found in the CD11b^hi^ subsets (Fig 3B). The double-negative subset was strongly positive for F4/80, while the CD11b^hi^Langerin^-^ subset was heterogenous for most markers analyzed. Both Langerin^+^ subsets displayed a similar phenotype with high expression of CD24, EpCam and CD64 (Fig 3B). Consistent with a previous study on Langerhans cells in the oral mucosa [26], we found tongue Langerin^+^ cells to be radiosensitive (S4A and S4B Fig). Moreover, immunofluorescent staining of tissue sections and epithelial sheets from infected animals revealed an intraepithelial localization of the Langerin^+^/MHCII^+^ cells in the tongue (S4C Fig).

As the phenotypical analysis did not allow categorizing the tongue CD11c^+^MHCII^+^ MNP populations into DCs and/or macrophages, we examined their expression of Zbtb46, a transcription factor selectively expressed by cDCs but no other myeloid and lymphoid cells [27]. The analysis of *Zbtb46*^*GFP*^ reporter mice in combination with EpCam expression as a surrogate for Langerin on the cell surface demonstrated that EpCam^+^ but not EpCam^-^ CD11c^+^MHCII^+^ cells were *bona fide* cDCs, whereas the EpCam^-^ subsets rather represented macrophages in the naïve mouse tongue (Fig 3C).

We then assessed the dependence of the tongue CD11c^+^MHCII^+^ MNPs on the transcription factors Batf3 and Klf4, which in other organs are lineage defining for cDC1s and a subset of cDC2s, respectivey [28,29]. Hematopoietic deletion of Klf4 had no impact on the tongue CD11c^+^MHCII^+^ MNPs (Fig 3D and 3E). In contrast, Batf3 deficiency resulted in a specific loss of the CD11b^low^Langerin^-^ subset in the tongue, which was surprising since those cells were Zbtb46-negative (Fig 3C). For comparison, spleen samples were analyzed in parallel confirming that CD11b^-^CD103^+^ cDC1s were clearly Batf3-dependent whereas CD11b^+^CD103^-^ cDCs were partially Klf4-dependent (Fig 3D and 3E). Together, our data revealed that the CD11c^+^MHCII^+^ compartment in the naïve tongue is unique and heterogenous with its Zbtb46^+^CD11b^+/low^Langerin^+^ DCs and Zbtb46^-^CD11b^+/low^Langerin^-^ macrophages that display an unprecedented onthological signature.

**Fig 4:**
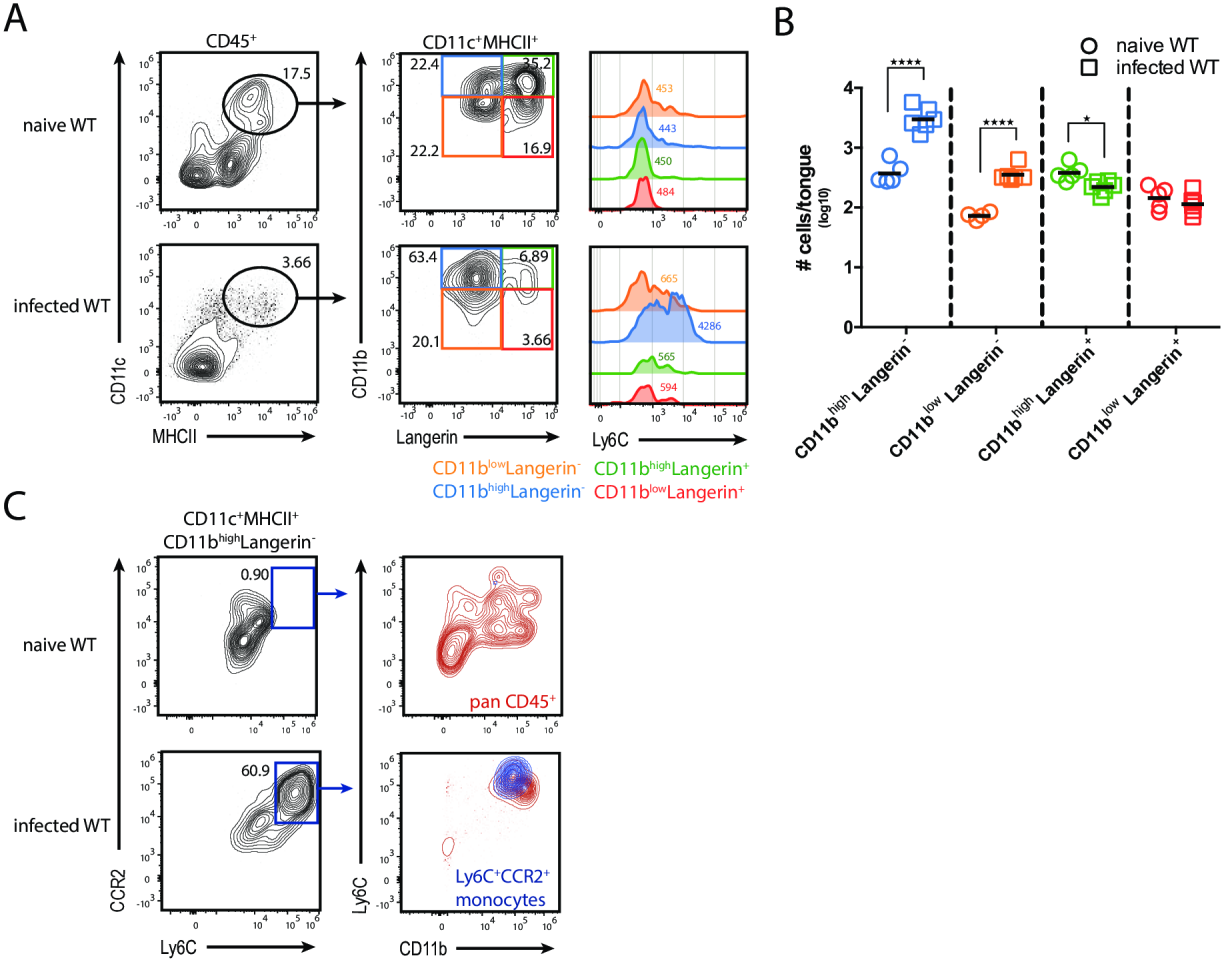
Dynamics of MNP subsets in the tongue during acute OPC. (**A**) Flow cytometric analysis of CD11c^+^MHCII^+^ MNP subsets in tongue of naïve and infected WT mice for the expression of Ly6C. Pre-gating is on CD45^+^ cells. Numbers in the histogram indicate MFI (median) of Ly6C expression. (**B**) Summary plot with absolute numbers of the CD11c^+^MHCII^+^ MNP subsets according to the analysis in **A** in naïve and infected tongue of WT animals. Each symbol represents one animal, the mean of each group is indicated. Graphs show pooled data from two independent experiments. (**C**) Analysis of CD11c^+^MHCII^+^CD11b^high^Langerin^-^ cells for Ly6C and CCR2 in the tongue of naïve and infected animals. An overlay of CD11c^+^MHCII^+^CD11b^+^Langerin^-^Ly6C^+^CCR2^+^ inflammatory monocytes (blue) onto all CD45^+^ cells (red) derived from naïve and infected WT mice is shown. Representative FACS plots are from one out of two independent experiments are shown. (*p<0.05; ****p<0.0001). See also S5 Fig.

Finally, we investigated the dynamics of these newly defined populations of tongue MNPs during OPC. The presence of *C*. *albicans* led to a rapid relocalization and clustering of intraepithelial MNPs in proximity of fungal hyphae (S5 Fig). The population of Langerin^+^ DCs was reduced in size on day 1 post-infection when compared to the naïve state (Fig 4A and 4B). The lack of Annexin-V^+^ staining suggested that the Langerin^+^ DCs were rather emigrating from the tongue epithelium than undergoing apoptosis (data not shown). The loss of Langerin^+^ cells was accompanied by an increase in Langerin^-^CD11c^+^MHCII^+^ MNPs in the tongue, which co-expressed Ly6C and CCR2, indicating that they were derived from Ly6C^high^ inflammatory monocytes (Fig 4C).

In conclusion, these data show that the tongue bears a complex network of MNPs with important tissue-specific pecularities and that acute OPC leads to dynamic changes within this network including the decline in Langerin^+^ DCs and the infiltration and differentiation of CD11b^+^Ly6C^high^ inflammatory monocytes.

### Multiple MNP subsets contribute to IL-1β and IL-6 production during acute OPC

Our observation that IL-1β and IL-6 are both important for induction of IL-17 during OPC prompted us to identify the cellular source(s) of these cytokines. We first assessed cytokine production by the two major myeloid cell populations in the infected tongue, namely CD11b^+^CD11c^+^MHCII^+^ MNPs (P1) and CD11b^+^CD11c^-^MHCII^-^ cells (P2), the latter comprise pre-dominantly neutrophils. Both populations contributed to the overall IL-1β production (S6A Fig), while—consistent with previous data [30]—no IL-1β was produced by the non-hematopoietic compartment (S6B Fig). Intracellular staining of pro-IL-1β served to identify IL-1β-producing cells. The specificity of the intracellular staining for pro-IL-1β was confirmed by applying the same staining panel to cells obtained from infected *Il1ab*^*-/-*^ mice (S6C Fig). In addition to IL-1β, the IL-1 receptor is also engaged by IL-1α, which is released by oral keratinocytes during OPC [30]. IL-6 on the other hand was produced by both, the hematopoietic and non-hematopoietic compartment in response to infection. Among the CD45^+^ cells, CD11b^+^CD11c^+^MHCII^+^ MNPs provided the main source of IL-6 with very little contribution of CD11b^+^CD11c^-^MHCII^-^ neutrophils (S6A, S6B and S6D Fig). Overall, our data defined the presence of multiple hematopoietic and non-hematopoietic cellular compartments providing IL-1β and IL-6 at the onset of OPC.

**Fig 5:**
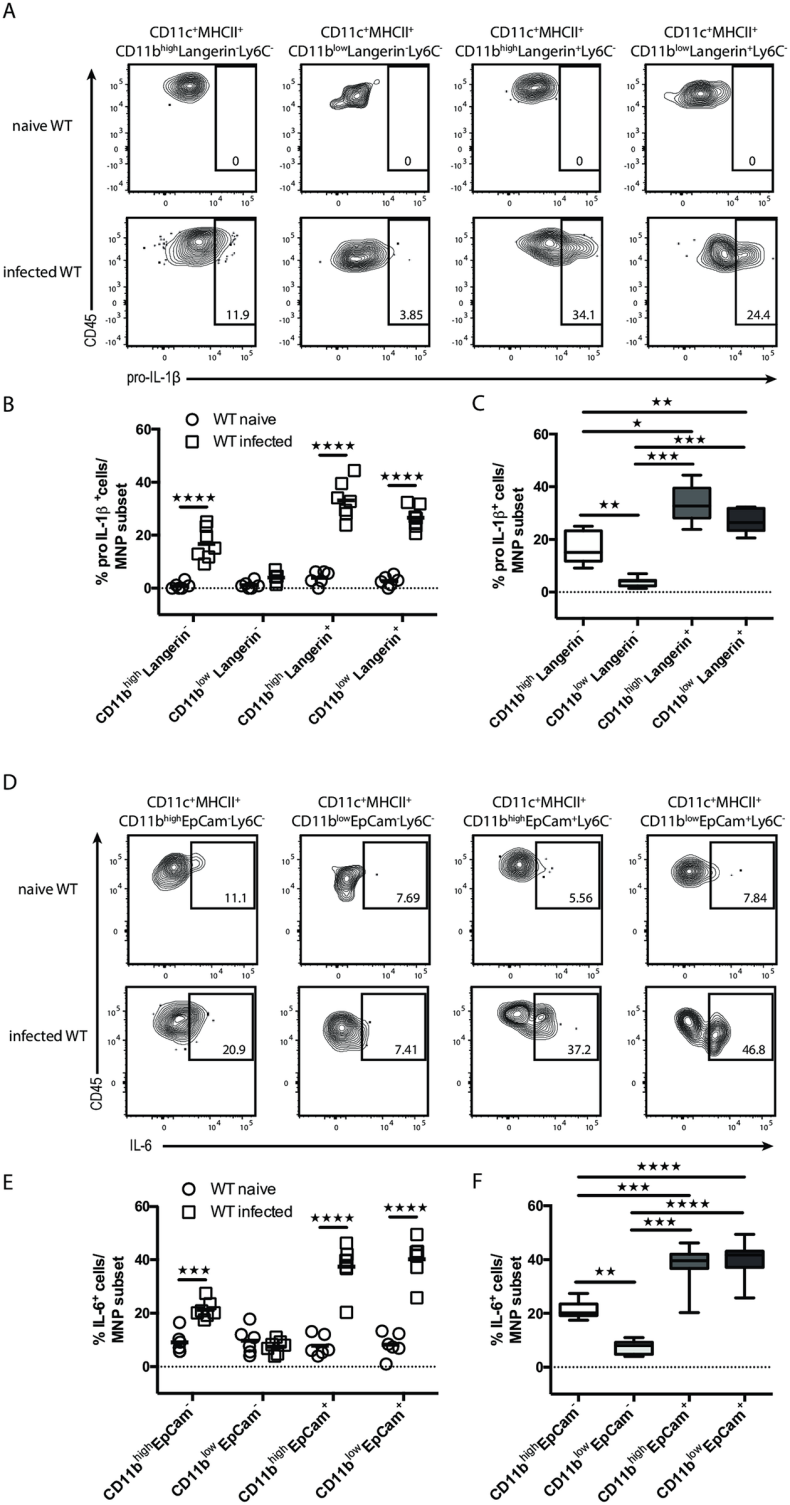
Multiple MNP subsets contribute to IL-1β and IL-6 production during acute OPC. Flow cytometric analysis of pro-IL-1β- (**A**-**C**) or IL-6-expressing CD11c^+^MHCII^+^MNP subsets (**D**-**F**) in naïve and infected WT mice. (**A**, **D**) Representative FACS plots. Pre-gating is on CD45^+^CD11c^+^MHCII^+^ cells. Numbers indicate the % of parent in each gate. (**B**, **E**) Summary plots with percentages of cytokine-expressing cells per MNP subset in naïve versus infected WT mice. Each symbol represents one animal, the mean of each group is indicated. (**C**, **F**) Summary graph comparing relative contribution of the four MNP subsets to the cytokine production in infected WT mice. Box-and Whisker plot shows mean of each group. Graphs in B, C, E and F show pooled data from two independent experiments. (*p<0.05; **p<0.01, ***p<0.001; ****p<0.0001.) See also S6 and S7 Figs.

Next, we aimed at investigating the contribution of individual MNP subset(s) to the overall production of IL-1β and IL-6 during acute OPC. Therefore, we combined our staining panel for the four MNP subsets (based on Langerin and CD11b expression) with intracellular cytokine staining for pro-IL-1β and IL-6 (S7A Fig). This revealed that pro-IL-1β and IL-6 were produced by three out of the four MNP subsets, namely Langerin^+^CD11b^+^ and Langerin^+^CD11b^-^ DCs as well as Langerin^-^CD11b^+^ MNPs (predominantly macrophages), while CD11b^-^Langerin^-^ MNPs did not produce either of the two cytokines (Fig 5A, 5B, 5D and 5E). Of all MNP subsets, the Langerin^+^ subsets displayed the highest proportion of IL-1β^+^ and IL-6^+^ cells (Fig 5C and 5F). Analyzing cytokine production by Ly6C^+^ inflammatory monocytes showed that these cells contributed only little to pro-IL-1β and not to IL-6 secretion (S7B and S7C Fig). In summary, our data show that tissue-resident MNPs, especially Langerin^+^ DCs, provide the IL-17A-inducing factors IL-1β and IL-6 during the onset of OPC.

### Langerin^+^ DCs and neutrophils provide IL-23 during acute OPC

**Fig 6:**
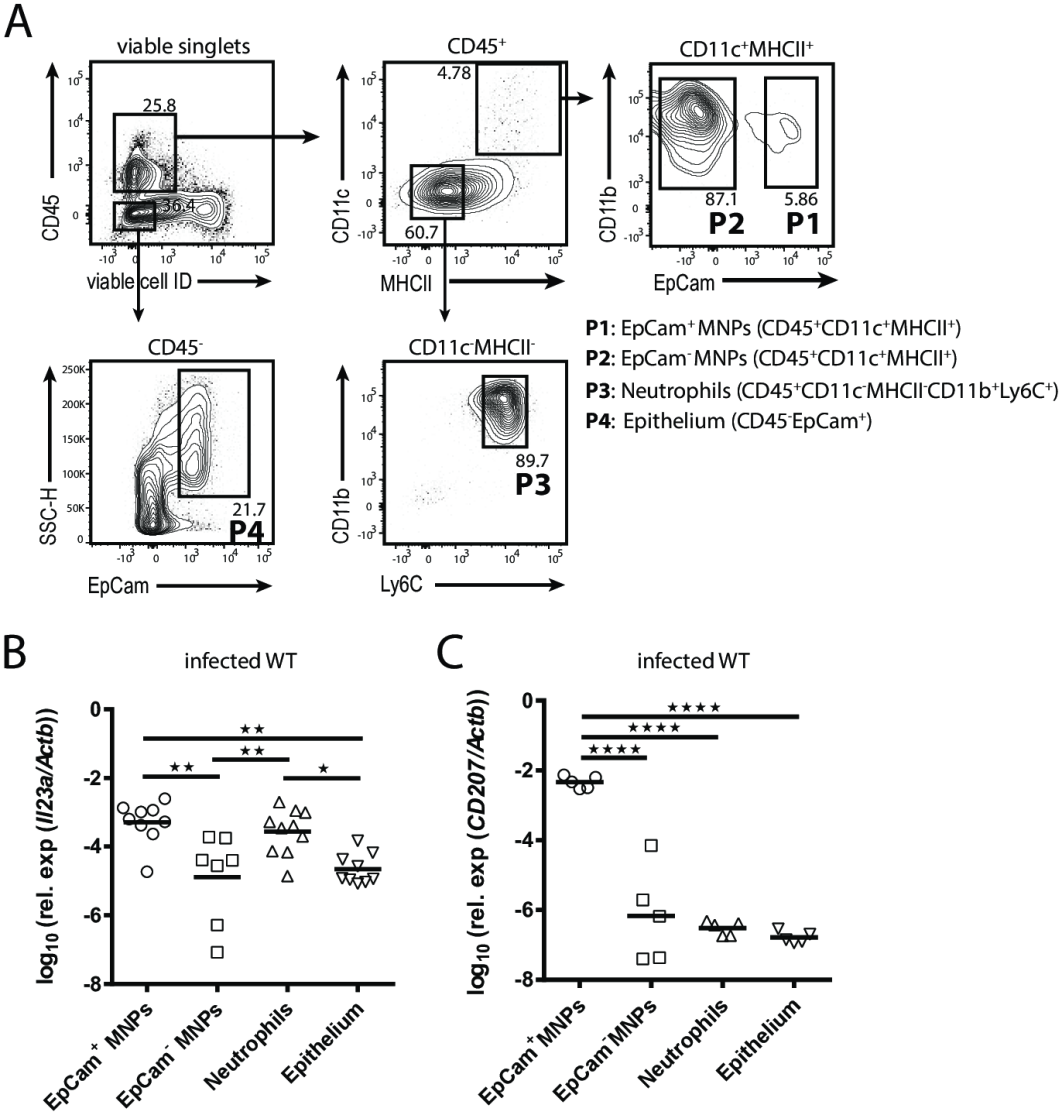
Langerin^+^ DCs and neutrophils provide IL-23 during OPC. (**A**) Representative gating strategy for sorting EpCam^+^ and EpCam^-^CD11c^+^MHCII^+^ MNPs, CD11c^-^MHCII^-^ CD11b^+^Ly6c^+^ cells and CD45^-^EpCam^+^ epithelial cells from the tongue of infected WT and *Il23a*^-/-^ mice. Pre-gating is on viable single cells. Numbers indicate the % of parent in each gate. (**B, C**) RT qPCR analysis of *Il23a* (B) and *CD207* expression (C) in the sorted cell populations of infected WT mice. Each symbol represents one animal, the mean of each group is indicated. Graphs show pooled data from two independent experiments (*p<0.05, **p<0.01, ****p<0.0001). See also S8 Fig.

Besides IL-1β and IL-6, IL-23 also contributes critically to innate IL-17A induction during OPC (Fig 2A and 2B). Determining IL-23 production at a cellular level remains difficult due to the lack of a functional antibody for detection of the specific cytokine subunit IL-23p19 by flow cytometry. To overcome this limitation, we FACS-sorted four major cell populations from infected tongues and quantified the expression of *IL23a* transcripts (coding for IL-23p19) by these cells by RT qPCR. For technical reasons we used EpCam instead of Langerin in the flow cytometry panel in this experiment. Our approach led to the identification of EpCam^+^CD11c^+^MHCII^+^ MNPs (corresponding to Langerin^+^ DCs) and CD11b^+^Ly6C^+^CD11c^-^MHCII^-^ cells (predominantly neutrophils) as the main sources of IL23p19, while EpCam^-^CD11c^+^MHCII^+^ MNPs (mostly macrophages) and CD45^-^EpCam^+^ epithelial cells contributed only marginally (Fig 6A and 6B). The specificity of the RT qPCR analysis for IL-23p19 was verified by analyzing IL-23p19 expression in the four cell populations sorted from infected *Il23a*^*-/-*^ mice (S8A Fig). The adequacy of using EpCam instead of Langerin was confirmed by analyzing *Cd207* transcript expression (coding for Langerin) in the sorted cell populations (Fig 6C). Overall our data demonstrate that EpCam^+^/Langerin^+^ DCs as well as neutrophils provide IL-23p19 at the onset of OPC.

We also examined expression of *Il1b* and *Il6* transcripts by the four sorted cell populations and could thereby confirm the results obtained by flow cytometry (Fig 5, S6 Fig), namely that IL-1β was expressed predominantly by EpCam^+^ MNPs, EpCam^-^ MNPs and neutrophils, and that IL-6 was expressed by EpCam^+^ DCs and to a lesser degree by EpCam^-^ macrophages (S8B and S8C Fig). Together, tissue-resident Langerin^+^/EpCam^+^ DCs thus stand out by their efficient expression of all three cytokines involved in innate IL-17A induction.

### Langerin^+^ DCs are critical for IL-17A production during acute OPC

After identification of multiple and partially overlapping myeloid cell subsets producing IL-17A-inducing cytokines, we aimed at evaluating their functional relevance for IL-17A production during infection. First, we targeted neutrophils, which represent the majority of the tongue-infiltrating leukocytes during actue OPC (S9A Fig) [15,30] and contribute to the overall IL-1β and IL-23p19 production in the infected tongue (Fig 6B, S6A Fig). We therefore treated mice with anti-Ly6G and anti-G-CSF antibodies to deplete neutrophils prior and during infection. This had only a limited impact on IL-17A production by the three CD90^+^ celluar subsets that did not reach statistical significance (S9B Fig). However, the actual contribution of neutrophils to the IL-17 response may likely be underestimated due to the difficulty to fully deplete these rapidly infiltrating cells from the infected tissue despite the administration of two distinct neutralizing/blocking antibodies [15,30]. Moreover, additional cellular sources, such as EpCam^+^ DCs, provide IL-17A-inducing cytokines that may compensate for the compromised neutrophil response.

**Fig 7:**
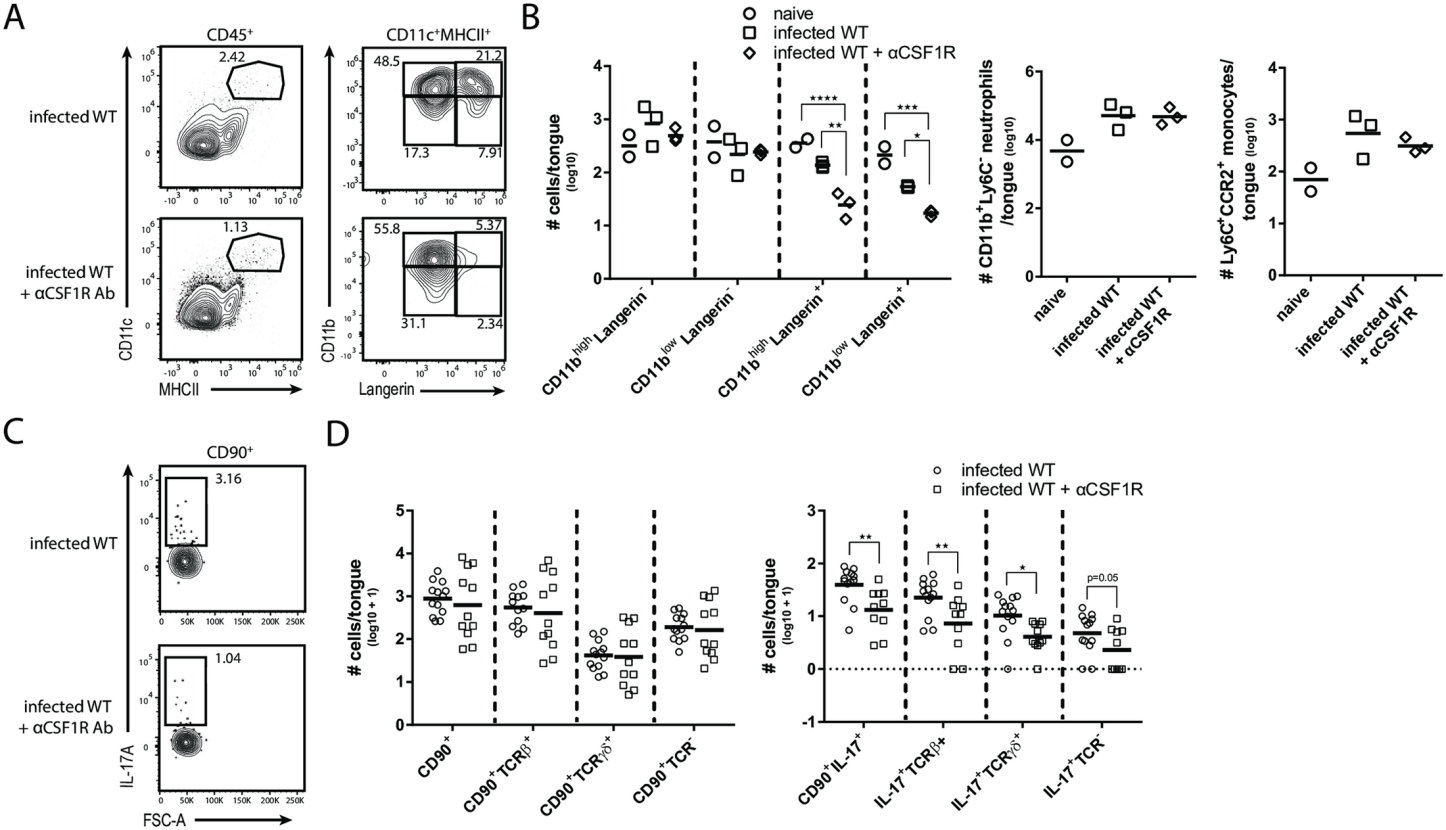
CSF1-dependent CD11c^+^MHCII^+^ DCs are critical for orchestrating IL-17A production by CD90^+^ cells during OPC. (**A**) Representative analysis of CD11c^+^MHCII^+^ MNPs in the tongue of infected WT animals with or without injection of αCSF1R antibody. Pre-gating is on CD45^+^ cells. **(B)** Summary plot with absolute numbers of CD11c^+^MHCII^+^ MNP subsets (according to the analysis in **A**, as well as absolute numbers of CD11c^+^MHCII^+^CD11b^+^Langerin^-^Ly6C^+^CCR2^+^ inflammatory monocytes and CD11c^-^MHCII^-^CD11b^+^Ly6C^-^ neutrophils from both experimental groups. Each symbol represents one animal, the mean of each group is indicated. Graphs display data representative for one out of two independent experiments. (**C**) Representative analysis of CD90^+^IL-17A^+^ cells in infected WT mice with or without anti-CSF1R antibody. (**D**) Summary plot with absolute numbers of CD90^+^ and CD90^+^IL-17A^+^ cells according to the analysis shown in **C**. Each symbol represents one animal, the mean of each group is shown. Graphs show pooled data form three independent experiments. (*p<0.05, **p<0.01, ***p<0.001). See also S9 Fig.

We also assessed the contribution of inflammatory monocytes to innate IL-17A induction during OPC, as these cells have previously been shown to promote the adaptive Th17 response to *C*. *albicans* in the oral mucosa [19]. However, infected *Ccr2*^*-/-*^ mice, in which CD11b^+^Ly6C^high^ monocytes recruitment to the infected tongue was strongly impaired (S9C and S9D Fig), displayed no defect in IL-17A production on day 1 post-infection by any of the three CD90^+^ cell subsets (TCRβ^+^, TCRγδ^+^, ILCs) compared to WT control mice (S9E Fig). Albeit not fully conclusive due to the incomplete monocyte depletion in *Ccr2*^*-/-*^ mice, these data are in line with our observation that Ly6C^+^ inflammatory monocytes contribute only weakly to the overall IL-1β production and do not produce IL-6 (S7C and S7E Fig), indicating that in contrast to the later phase of OPC, inflammatory monocytes are dispensible for the early IL-17A response during acute OPC. Finally, we examined the role of the newly identified tongue MNP subsets in the initiation of innate IL-17A production during OPC. For this, we analyzed different genetic and antibody-depletion models with selective MNP defects. The lack of the CD11b^low^Langerin^-^ MNP population in *Batf3*^*-/-*^ mice (Fig 3D and 3E) had no impact on the number of IL-17A-producing CD90^+^ leukocytes during acute OPC (data not shown), which is in line with a previous publication showing that Batf3-deficiency in mice is dispensable for IL-17-mediated antifungal defense during OPC [31]. The same was true for IL-17A production in *VAV*^*Cre*^*KLF4*^*fl/fl*^ animals (data not shown), which was not surprising given that these mice did not display any changes in the tongue MNP network (Fig 3D and 3E). Homeostasis of tissue-resident MNPs, including macrophages and Langerin^+^ DCs, depends on CSF1R signaling and consequently these cells can be depleted *in vivo* upon antibody-mediated blockade of the CSF1R [32,33]. We thus aimed at depleting tongue CD11c^+^MHCII^+^ MNPs by administering an anti-CSF1R blocking antibody prior to OPC onset. This resulted in the selective loss of Langerin^+^CD11c^+^MHCII^+^ DCs, while Langerin^-^CD11c^+^MHCII^+^ MNPs, Ly6C^+^CCR2^+^ monocytes or neutrophils were not significantly affected by the treatment (Fig 7A and 7B). Importantly, the loss of Langerin^+^ DCs in the tongue was accompanied by a significant reduction in IL-17A-production by all three CD90+ subsets if compared to non-treated WT control mice (Fig 7C and 7D). These results were in line with our findings of Langerin^+^ DCs being the only tissue-resident MNP population in the tongue producing all three IL-17A-inducing cytokines IL-1β, IL-6 and IL-23 (Figs 5 and 6) and demonstrate that Langerin^+^ DCs are key players in the IL-17 response during acute OPC.

## Discussion

The cytokine IL-17 has gained much attention due to its association with auto-inflammatory disorders such as psoriasis, psoriatic arthritis or ankylosing spondylitis [34,35] and the remarkable success in treating these diseases with IL-17 targeting reagents [36,37]. However, IL-17 is also crucial for mediating immune homeostasis in barrier tissues that are continuously exposed to microbes. Over the past decade, the lower gastrointestinal tract has been intensively studied in this context [38–40]. IL-17 production in the gut is determined by the microbiota [41–43] and the Gram positive segmented filamentous bacterium SFB has been identified as a major driver of the response [44,45]. The regulation of IL-17 in other mucosal tissues than the gut is less well studied. Only recently, it was shown that the homeostatic Th17 response in the gingiva is independent of the microbiota but rather a consequence of constant tissue damage elicited by mastication [46].

IL-17 immunity plays an essential role in host defense against opportunistic infections with the fungal pathogen *C*. *albicans* as evidenced by primary immunodeficiency patients with defects in genes of the IL-17 pathway that suffer from chronic mucocutaneous candidiasis. One of the tissues most frequently affected by *C*. *albicans* is the oral cavity. Experimental OPC in mice triggers prominent IL-17 induction and fungal clearance depends on the rapid induction of IL-17 in the infected mucosa during the onset of infection [13]. Here, we demonstrated that upon OPC in mice, IL-17 is produced by a tripartite population of CD90^+^ leukocytes in the tongue, comprising αβ T cells, γδ T cells and ILCs. Production of innate IL-17 is under the control of CSF1-dependent Langerin^+^ DCs, which are the major source of the IL-17-inducing cytokines IL-1β, IL-6 and IL-23 in the oral mucosa.

Earlier work from our group already proposed ILCs to provide IL-17A at the onset of OPC in experimentally infected mice [13]. This observation was based on the rapid kinetics of IL-17 induction and the fact that *Rag1*^*-/-*^ but not *Rag1*^*-/-*^*Il2rg*^*-/-*^ or anti-CD90-treated *Rag1*^*-/-*^ mice were protected from infection due to their capacity of upregulating IL-17 in the infected mucosa. Furthermore, MHC-II-deficiency did not impair IL-17A expression in the infected mucosa indicating that conventional MHCII-mediated antigen presentation is dispensable for IL-17A production during OPC [13]. However, our findings were challenged by the work from Conti et al., who reported IL-17 expression by RAG-dependent lymphocytes, foremost αβ and γδ T cells, during OPC [20]. These seemingly contradictory results have arisen not least because of indirect assessments of IL-17A production during infection by either measuring *Il17a* and *Il17f* transcripts in crude tongue extracts [13] or monitoring IL-17A promoter activity in *Il17a*^*eYFP*^ fate reporter mice [20]. Here, we reconcile the discrepancy and demonstrate the existence of three separate and complementary IL-17-producing cell types by direct visualization of IL-17A and IL-17F cytokines in the infected tongue. These three cellular subsets act in an at least partially redundant manner: selective lack of αβ or γδ T cells does not affect fungal control and only deletion of all three subsets phenocopies the high susceptibility of IL-17RA or IL-17RC-deficient mice to OPC [11,13,15], underlining the robustness of the IL-17 response to the fungus.

Based on their independence of RAG and their expression of RORγt, the TCR-negative IL-17 producers are part of the family of group 3 ILCs, although they lack expression of CCR6, NCR1 and MHCII, which are characteristic of at least some ILC3s [47,48]. Visualization of IL-17A and IL-17F protein expression by flow cytometry not only allowed us to define the sources of IL-17 but also offered the opportunity to investigate the regulatory mechanisms of IL-17 production during acute OPC. We confirmed the critical role of IL-23 for innate IL-17 induction, an observation that is in line with previous work demonstrating that *Il23a*^*-/-*^ mice phenocopy *Il17ra*^*-/-*^ and *Il17rc*^*-/-*^ mice in their inability to clear *C*. *albicans* [11,13]. However, it also became evident that the defect in IL-17 production in response to OPC was not complete in *Il23a*^*-/-*^ animals, indicating that IL-23 may have (an) additional IL-17-independent function(s) in antifungal defense. Moreover, IL-23 may share redundancy with other IL-17-inducing cytokines. The dependence on IL-23 was not equally pronounced for all IL-17-producing subsets, suggesting that the relative contribution of different cytokines to IL-17 induction may differ for different cellular sources. In addition, the technical limitations of the experimental system due to the small cell numbers recoverable from the tongue may also mask clearer associations.

Both, IL-1 and IL-6 have been implicated in the regulation of IL-17 immunity in the gut [49,50] and IL-6 was also implicated in Th17 polarization in the gingiva during steady state [46]. Here, we report that these cytokines also trigger innate IL-17 production in response to *C*. *albicans* in the oral mucosa. The impact of IL-1 and IL-6 on innate IL-17 production was somewhat overlooked before when each pathway was examined in isolation [13,20]. We now demonstrate that only concurrent blockade of both IL-1 and IL-6 pathways resulted in a significant drop of IL-17 induction in infected mice.

Tongue-resident MNP populations have not been characterized in detail in mice. Our phenotypic and transcription factor analysis of CD11c^+^MHCII^+^ MNPs revealed the presence of two heterogeneous populations of Zbtb46^-^Langerin^-^ macrophages and Zbtb46^+^Langerin^+^ DCs in the naïve tongue. Whether the Langerin^+^ DCs are *bone fide* Langerhans cells or represent the mucosal analogue of Langerin^+^ dermal DCs remains to be determined [51]. In terms of their phenotype and *in situ* localization they closely resemble Langerhans cells in the gingiva and the buccal mucosa [26]. Moreover, their independence of Batf3 further supports that they are indeed Langerhans cells [52]. Langerhans cells in the oral mucosa have been shown to differ from their skin counterparts in terms of their ontogeny, as they are derived from circulating radiosensitive precursors instead of radio-resistant embryonic precursors [26] and we confirmed this to be the case in the tongue. That MNPs in the tongue are different from MNPs in other tissues is also exemplified by the Langerin^-^ MNP populations. We found the CD11b^low^Langerin^-^ subset to depend on Batf3, a lineage-determining transcription factor for cDC1s, but at the same time to lack Zbtb46 expression, thus calling into question whether it constitutes a Batf3-dependent subset of macrophages or a special population of Zbtb46-independet DCs. While future work will be needed to fully clarify the ontogeny of all four oral MNP subsets, our detailed dissection of the MNP network in the murine tongue sets the stage for interrogating the contribution of individual populations to immune homeostasis and defense.

Among the accessory cells supplying IL-1 and IL-6 during acute OPC, we identified non-hematopoietic cells, which have been shown before to release IL-6 and IL-1α in the oral mucosa of mice [30]. In addition, we found (several) complementary myeloid cell populations serving as hematopoietic sources of IL-1, IL-6 and/or IL-23 during infection. Neutrophils that rapidly infiltrate to the site of infection were found to produce IL-1β and IL-23 during OPC. While neutrophils can themselves serve as a source of IL-17A under certain circumstances [53,54], we have no evidence for IL-17 production by neutrophils during OPC. Conversely, neutrophils may support IL-17 production by CD90^+^ leukocytes as they secrete IL-17-promoting cytokines during infection in the oral cavity. Macrophages and monocytes (defined as Langerin^-^ MNPs) contributed to the production of IL-1β and IL-6, but did not produce IL-23. Langerin^+^ MNPs however were the only cellular subset producing all three IL-17-inducing factors. Together with their strategic location in the outermost layer of the epithelium at the onset of the infection, prior to the arrival of infiltrating inflammatory cells, this unique property predisposes Langerin^+^ DCs as the primary IL-17-inducing cellular subset. Targeting MNPs via antibody-mediated blockade of CSF1R confirmed the crucial role of Langerin^+^ cells as the primary cellular determinant for IL-17 induction at the onset of OPC. Langerin^+^ DCs have been implicated in IL-17-mediated immunity against *C*. *albicans* previously: during experimental cutaneous candidiasis, the constitutive absence of Langerhans cells in huLangerin-DTA mice [55] resulted in a drastric reduction in Th17 differentiation in skin-draining lymph nodes [56]. Reminiscent of experimental OPC, infection of mice with *C*. *albicans* via the epicutaneous route also triggered an immediate local IL-17 response within one day of infection, which is dominated by γδ T cells [12]. The release of IL-17 by γδ T cells in the skin during epicutaneous infection was not dependent on Langerhans cells, but rather on CD301b^+^ dermal DCs [12]. In the oral mucosa however and in contrast to the skin, adaptive immunity against *C*. *albicans* does not rely on Langerin^+^ cells, but instead depends on CCR2-dependent inflammatory DCs and other Flt3-dependent migratory DCs [19]. Therefore, the contribution of specific MNP subsets to the regulation of IL-17 production in different epithelial tissues and in different phases during infection emphasizes the dynamic and tissue-specific regulation of IL-17 immunity to *C*. *albicans*. Here, we revealed a novel role of Langerin^+^ DCs in the tongue coordinating the acute IL-17 response during OPC.

## Materials and methods

### Ethics statement

All mouse experiments in this study were conducted in strict accordance with the guidelines of the Swiss Animals Protection Law and were performed under the protocols approved by the Veterinary office of the Canton Zurich, Switzerland (license number 201/2012 and 183/2015). All efforts were made to minimize suffering and ensure the highest ethical and humane standards.

### Animals

WT C57BL/6j mice were purchased by Janvier Elevage. *Il1r*^*-/*-^ [57], *Rag1*^*-/*-^ [58,59], *Ccr2*^*-/-*^ [60], *Rorc*^*Cre*^ [61], *Il23p19*^*-/-*^ [62], *Il17af*^*-/-*^ [63] (a kind gift from Immo Prinz, MH Hannover, Germany), *Rorc*^*Cre*^*R26R*^*eYFP*^ x *Rag1*^*-/-*^ (a kind gift from Burkhard Becher, University of Zurich, Switzerland) and *Rosa26r*^*eYFP*^ animals [64] were bred at the Institute of Laboratory Animals Science (University of Zurich, Zurich, Switzerland). *Klf4*^*fl/fl*^, *Vav*^*Cre*^*Klf4*^*flfl*^ [65], *Batf3*^*-/-*^ [28] and *Zbtb46*^*GFP/+*^ [27] animals were bred at the Department of Biomedicine, University of Basel, Switzerland. *Il1ab*^*-/-*^ mice were obtained from Wolf-Dietrich Hardt, ETH Zurich, Switzerland. All mice were on the C57BL/6 background except for *Batf3*^*-/-*^animals, which were on mixed Sv129/B6 background. The animals were kept in specific pathogen-free conditions and used at 6–12 weeks of age in sex- and age-matched groups.

### Fungal strain and infection

The *C*. *albicans* strain SC5314 [66] was used for all experiments if not stated otherwise. CAF-yCherry was obtained from Robert Wheeler [67]. Mice were infected with 2.5x10^6^ cfu of *C*. *albicans* sublingually as described [68] without immunosuppression. Mice were monitored for morbidity and euthanized in case they showed severe signs of pain or distress.

### Isolation of tongue and spleen cells

All analyses of infected animals in this study were carried out at 24 hours post infection. Mice were anaesthetized with a sublethal dose of Ketamin (100mg/kg), Xylazin (20mg/kg) and Acepromazin (2.9mg/kg) and perfused by injection of PBS into the right heart ventricle prior to removing the tongue and/or the spleen. For most experiments except for the analysis of tongue-resident MNPs, we isolated leukocytes as previously described in detail [69]. Briefly, tongues were cut into fine pieces and digested with DNase I (200μg/ml) and Collagenase IV (4.8 mg/ml, Invitrogen) in PBS for 50 minutes at 37°C. Single cell suspensions were obtained by passing the digested tissue through a 70μm strainer using ice-cold PBS supplemented with 1% FCS and 2mM EDTA. Tongue leukocytes were enriched over a 40% Percoll gradient before they were stained for flow cytometry. For the characterization of tongue-resident MNPs, tongues were cut in half and the underlying muscle tissue was carefully removed using a scalpel. The remaining tongue tissue was cut into fine pieces and digested with Trypsin (1mg/ml), DNase I (200mg/ml) and Collagenase IV (2.4mg/ml) in PBS for 45 minutes at 37°C. Single cell suspensions were obtained by passing the digested tissue through a 70μm strainer using ice-cold PBS supplemented with 1% FCS and 2mM EDTA and then stained for flow cytometry.

### Immunofluorescence of tongue sections and epithelial sheets

For tongue sections, the tissue was embedded in Tissue-Tek OCT compound (Sakura) and snap-frozen in liquid nitrogen. Sagittal cryo-sections were cut at a thickness of 9 μm with a HM525 Microtome Cryostat and were mounted to super frost glass slides (Thermo Scientific). The specimen were allowed to dry at room temperature for 30min prior to immunofluorescence staining. For epithelial sheets, the tongue was cut longitudinally and the muscle tissue was carefully removed with a scalpel. The tissue was placed with the epithelial layer upwards onto a Dispase II solution (2.85 mg/ml PBS, Roche) and incubated for 1 hour at 37°C. Epithelial sheets were obtained by separating the lamina propria from the epithelium using two watchman tweezers. For immunofluorescence staining the specimen were fixed either with methanol at 20°C for 20 minutes or with acetone a room temperature for 10 minutes depending on the antibody used for the staining. The following antibodies were used: anti-Langerin (clone 929F.3, hybridoma supernatant), anti-MHCII (clone M5/114.15.2, Biolegend) and anti-CD11c (clone HL3, BD Bioscience). The stained specimens were mounted with Mowiol and stored at 4°C. Images were acquired with a digital slide scanner (NanoZoomer 2.0-HT, Hamamatsu) and analyzed with NDP.view2.

### *In vivo* administration of antibodies and Brefeldin A

To block cytokine secretion, infected mice were treated with Brefeldin A (Axon Lab AG, 250μg per mouse i.p.) three hours prior to euthanization. For IL-6 neutralization, animals were injected with anti-IL-6 (clone MP5-20F3, BioXCell, 60μg per mouse i.p.) directly after infection and again eight hours later. For neutrophil depletion, mice were treated with anti-Ly6G (clone 1A8, BioXCell, 150μg per mouse i.p.) on day -1 and with anti-G-CSF (clone 67604, R&D Systems, 10μg per mouse i. p.) on day -1 and day 1 post-infection. Anti-CSF1R (clone AFS98, BioXCell or produced and in-house and obtained from M. Greter) was injected on day -3 (2mg per mouse i. p.), on day -1 (0.5mg per mouse i. p.) and day 0 (0.5mg per mouse i. p.) of infection.

### Flow cytometry

All antibodies were from BioLegend, if not stated otherwise. For Flow cytometric analysis, single cell suspensions of the tongue and the spleen were stained in PBS supplemented with 1% FSC, 5mM EDTA and 0.02% NaN_3_. LIVE/DEAD Near IR stain (Life Technologies) was used for exclusion of dead cells. The following antibodies were used for surface markers: anti-CD90.2 (30-H12), anti-CD45.2 (104), anti-TCRβ (H57-597), anti-TCRγδ (GL3), anti-CD11b (eBioscience, M1/70), anti-CD11c (N418), anti-MHCII (M5/114.15.2), anti-CD3 (145-2C11), anti-CCR6 (29-2L17), anti-CD24 (M1/69), anti-NCR1 (29A14), anti-CD122 (TM-β1), anti-CD44 (IM7), anti-CD69 (H1.2F3), anti-ki67 (16A8), anti-Ly6C (HK1.4), anti-CCR2 (SA203G11), anti-CD64 (X54-5/7.1), anti-Langerin (929F3), anti-EpCam (G8.8), anti-Ly6G (1A8), anti-XCR1 (ZET), anti-Sirpα (P84), anti-CD103 (2E7). For intracellular cytokine staining, tongue cells were fixed and permeabilized using BD Cytofix/Cytoperm reagent (BD Bioscience) and subsequently incubated in Perm/Wash buffer (BD Bioscience) containing the following cytokine-directed antibodies or the respective isotype controls: anti-IL-17A (TC11-18H10.1), anti-IL-17F (8F5.1A9), anti-pro-IL-1β (NJTEN3) and anti-IL6 (MP5-20F3). CD1d surface expression was stained with anti-CD1d tetramers (Proimmune). All extracellular and intracellular staining steps were carried out on ice. Cells were acquired on a FACS LSR II Fortessa (BD Biosciences) or on a FACS Gallios (Beckman Coulter) and the data were analyzed with FlowJo software (Tristar). In all the experiments, the cells were pre-gated on viable and single cells for analysis. Absolute cell numbers of CD90^+^ and CD90^+^IL-17A^+^ cells and their respective subpopulations were calculated based on a defined number of counting beads (BD Bioscence, Calibrite Beads), which were added to the samples before flow cytometric acquisition.

### FACS-sorting, RNA isolation and RT qPCR

For sorting cells from the infected tissue, single cell suspensions of the tongue were stained in PBS, supplemented with 1% FSC and 5mM EDTA, using the same antibodies as described in the previous section. Using a FACS Aria III, 50–100 target cells per defined population were sorted per well of a 96-well plate (Eppendorf) containing RLT Plus RNeasy^®^ lysis buffer (Qiagen). Lysates were snap-frozen and stored at -80°C until further processing. Whole-transcriptome amplification was performed following the Smart-Seq2 protocol [70]. Briefly, Agencourt RNAClean XP paramagnetic beads (Beckman Coulter) in combination with a DynaMag-96 side skirted magnet (Thermo Fisher) were applied to purify whole-genome RNA. Subsequently cDNA was generated using the SuperScript II Reverse Transcriptase Kit (Thermo Fisher), and further amplified with HiFi HotStart PCR Mix (KAPA Biosystems). For DNA clean-up, Agencourt AMPure XP beads (Beckman Coulter) were used as above. Optimal DNA concentration for real-time qPCR assays was determined by testing sample serial dilution for the expression of the control gene *Actb*. RT qPCR was performed using SYBR Green (Roche) and a QuantStudio 7 Flex (Life Technology) instrument. The primers were *Actb* fwd 5´-CCCTGAAGTACCCCATTGAAC-3´, *Actb* rev 5´-CTTTTCACGGTTGGCCTTAG-3´; *Il1b* fwd 5´-TACAGGCTCCGAGATGAACA-3´, *Il1b* rev 5´-AGGCCACAGGTATTTTGTCG-3´; *Il6* fwd 5´-GAGGATACCACTCCCAACAGACC-3´, *Il6* rev 5´-AAGTGCATCATCGTTGTTCATACA-3´, *Il23a* fwd 5´- CCAGCAGCTCTCTCGGAATC-3´, *Il23a* rev 5´-TCATATGTCCCGCTGGTGC-3´; *Cd207* fwd 5´-ATGTTGAAAGGTCGTGTGGAC-3´, *Cd207* rev 5´- GTGGTGTTCACTATCTGCATCT-3´; All qRT-PCR assays were performed in duplicates and the relative expression (rel. expr.) of each gene was determined after normalization to *Actb* transcript levels.

### Statistics and data transformation

Cell numbers of CD90^+^ and CD90^+^IL-17A^+^ cells and their respective subpopulations were transformed using the formula Y = y(Log10+1) to plot absolute cell numbers including 0 values. Bar of each data set indicate arithmetic mean. Statistical significance was determined by unpaired Student’s-test with Holm-Sidak correction for multiple comparison, one-way or two-way ANOVA with Tukey’s multiple comparison test using GraphPad Prism software with *p< 0.05; **p<0.01; ***p<0.001; ****p<0.0001.

## Supporting information

S1 Fig Identification of IL-17A-producing lymphocytes in the murine tongue by intracellular staining and flow cytometry. (**A**) Gating strategy for identifying CD90^+^IL-17A^+^ cell populations in the tongue of infected mice. (**B**) Analysis of CD90^+^IL-17A^+^ cells in the tongue of infected WT and *Il17af*^*-/-*^ animals. (**C**) Analysis of IL-17A and IL-17F co-expression by CD90^+^ cells in infected WT animals. (**D**) Analysis of IL-17A and CD1d expression by CD90^+^ cells in infected WT animals. Data shown in **B-D** are representative of one out of two independent experiments. Pre-gating is on CD45^+^ cells. Numbers indicate the % of parent in each gate.
(TIF)

S2 Fig Phenotype of IL-17A-producing cells during acute OPC. (**A**) Flow cytometric analysis of the TCRβ^+^, TCRγδ^+^ and TCR^-^ subsets for the indicated markers. Pre-gating is on CD45^+^CD90^+^ (upper panels) or CD45^+^CD90^+^IL-17A^+^ cells (lower panels). Representative plots from one out of two or three independent experiments are shown. Numbers indicate the % of cells in the gate.
(TIF)

S3 Fig IL-1 and IL-6 play a redundant role for IL-17 production. IL-17A^+^ cellular subsets in the tongue of infected WT and *Il1ra*^*-/-*^ animals (**A**-**B**) or IL-17A^+^ cellular subsets in the tongue of infected WT mice that were treated with anti-IL-6 antibody or left untreated (**C**-**D**). (**A**, **C**) Representative plots. Pre-gating is on CD45^+^CD90^+^ cells. Numbers indicate the % of parent in each gate. (**B, D**) Summary graphs with the total CD90^+^ and CD90^+^IL-17A^+^ cells as well as the respective TCRβ^+^, TCRγδ^+^ and TCR^-^ subsets according to the analysis shown in **A** and **C**. Each dot represents one animal, the mean of each group is indicated. Graphs show pooled data from three independent experiments.
(TIF)

S4 Fig Characterization of tongue-resident MNP subsets in naïve animals. (**A**) Experimental scheme for bone marrow chimera to investigate radio-resistance of tongue-resident CD11c^+^MHCII^+^ MNPs. (**B**) Flow cytometric analysis of tongue-resident CD11c^+^MHCII^+^ MNP subsets for the expression of CD45.1 and CD45.2 in naïve, reconstituted WT animals. Representative plots from one out of two independent experiments are shown. Numbers indicate the % of cells in the gate. (**C**) Microscopy analysis of MNPs for their expression of Langerin and CD11c in sagittal sections (left, scale bar = 100μm) and for MHCII in epithelial sheets of the tongue from naïve WT mice (right, scale bar = 100μm (top) and 30μm (bottom)). Representative images from one out of two independent experiments are shown.
(TIF)

S5 Fig Dynamic changes in the MHCII^+^ MNP network upon OPC. WT mice were infected with yCherry-expressing *C*. *albicans* strain CAF-yCherry. Microscopy analysis of *C*. *albicans* (red) and MHCII^+^ MNPs (green) in epithelial sheets obtained from naïve and infected WT mice. Representative pictures from one out of two independent experiments are shown (scale bar = 100μm).
(TIF)

S6 Fig Hematopoietic and non-hematopoietic cells provide IL-1β and IL-6 during acute OPC. (**A**) Expression of pro-IL-1β and IL-6 was analyzed by flow cytometry in CD11c^+^MHCII^+^ (P1) and CD11c^-^MHCII^-^ populations (P2) in the tongue of naïve and infected WT mice. Pre-gating is on CD45^+^ cells. (**B**) Flow cytometric analysis of CD45^-^ tongue cells from naïve and infected WT mice for pro-IL-1β and IL-6 expression. Pre-gating is on CD45^-^ cells. (**C**) The specificity of the pro-IL-1β staining was verified by comparing the antibody staining of cells from infected WT with infected *Il1ab*^*-/-*^ animals. Pre-gating is on CD45^+^ cells. (**D**) The specificity of the IL-6 staining was assessed by means of an isotype control antibody as shown for CD45^-^ cells. Numbers indicate the % of parent in each gate. Representative plots from one out of two independent experiments with 2 animals each are shown in each panel.
(TIF)

S7 Fig Flow cytometric analysis of IL-1β and IL-6 expression in CD11c^+^MHCII^+^ MNP subsets in the tongue during acute OPC. (**A**) Gating strategy for identifying pro-IL-1β-expressing cells in CD11c^+^MHCII^+^ MNP subsets in the tongue of infected WT mice. Cells were gated on viable CD45^+^ cells. Numbers indicate the % of cells in the gate. The same gating strategy was used to identify IL-6-expressing cells in CD11c^+^MHCII^+^ MNP subsets. (**B—E**) Flow cytometric analysis and summary graphs of pro-IL-1β or IL-6 expression in Ly6C^+^ inflammatory monocytes in the tongue of naïve and infected WT animals. (**B**, **D**) Representative flow cytometric analysis of pro-IL-1β (B) or IL-6 (D) expression in CD11c^+^MHCII^+^CD11b^high^Ly6C^+^ and CD11c^+^MHCII^+^CD11b^low^Ly6C^+^ inflammatory monocytes in the tongue of naïve and infected WT animals. Representative plots are from one out of two independent experiments. Numbers in the dot plots indicate the % of cells in the gate and are summarized in the graph. (**C, E**) Summary plots with percentages of cytokine-expressing monocytes in naïve versus infected WT mice. Each symbol represents one animal, the mean of each group is indicated. Data are pooled from two independent experiments. (***p<0.001).
(TIF)

S8 Fig Quantification of IL-1β and IL-6 transcripts in sorted tongue cell populations. (**A, B**) RT qPCR analysis of *Il23a* (A), *Il1b* (B, left) and *Il6* (B, right) expression in sorted CD11c^+^MHCII^+^EpCam^+^ MNPs, CD11c^+^MHCII^+^EpCam^-^ MNPs, neutrophils and epithelial cells from infected WT (B) or *Il23a*^*-/-*^ mice (A). Each symbol represents one animal, the mean of each group is indicated. Data are pooled from two independent experiments (**p<0.01, ***p<0.001, ****p<0.0001).
(TIF)

S9 Fig Effect of neutrophils and monocyte deficiency on IL-17 production. (**A**) Gating strategy for identifying myeloid cell populations in the tongue of infected mice, including CD90^-^CD11c^+^MHCII^+^ MNPs, CD11c^-^MHCII^-^CD11b^+^Ly6G^+^ neutrophils and CD11c^-^MHCII^-^CD11b^+^Ly6C^high^ monocytes. Numbers indicate the % of cells in the gate. Numbers in the histograms in **A** indicate MFI (median) of Ly6C. (**B**) Summary plot with absolute numbers of CD90^+^ and CD90^+^IL-17A^+^ cells and the respective TCRβ^+^, TCRγδ^+^ and TCR^-^ subsets isolated from the tongue of infected WT mice that were or were not treated with anti-G-CSF and anti-Ly6G antibodies. Each dot represents one animal. The mean of each group is indicated. Graphs show pooled data from three independent experiments. (**C—E**) Flow cytometric analysis of Ly6C^+^Ly6G^-^ monocytes in the tongue of infected WT and *Ccr2*^*-/-*^ mice. Pre-gating is on CD45^+^CD90^-^CD11c^-^MHCII^-^CD11b^+^ cells. Representative FACS plots are shown in **C** and summary graph with absolute numbers of CD11b^+^Ly6C^+^Ly6G^-^ monocytes is shown in **D**. Each symbol represents one animal, the mean of each group is indicated. Data are from one out of two independent experiments. (**E**) Absolute numbers of CD90^+^ and CD90^+^IL-17A^+^ cells and the respective TCRβ^+^, TCRγδ^+^ and TCR^-^ subsets in the tongue of infected WT or *Ccr2*^*-/-*^ mice. Each symbol represents one animal, the mean of each group is indicated. Graphs show pooled data form three independent experiments. (***p<0.001).
(TIF)
