# Supplementary figures and images for: Langerin+ DCs regulate innate IL-17 production in the oral mucosa during Candida albicans-mediated infection
Source: PLoS Pathog. 2018 May 21;14(5):e1007069. doi: 10.1371/journal.ppat.1007069 (PMC5983869; doi:10.1371/journal.ppat.1007069)

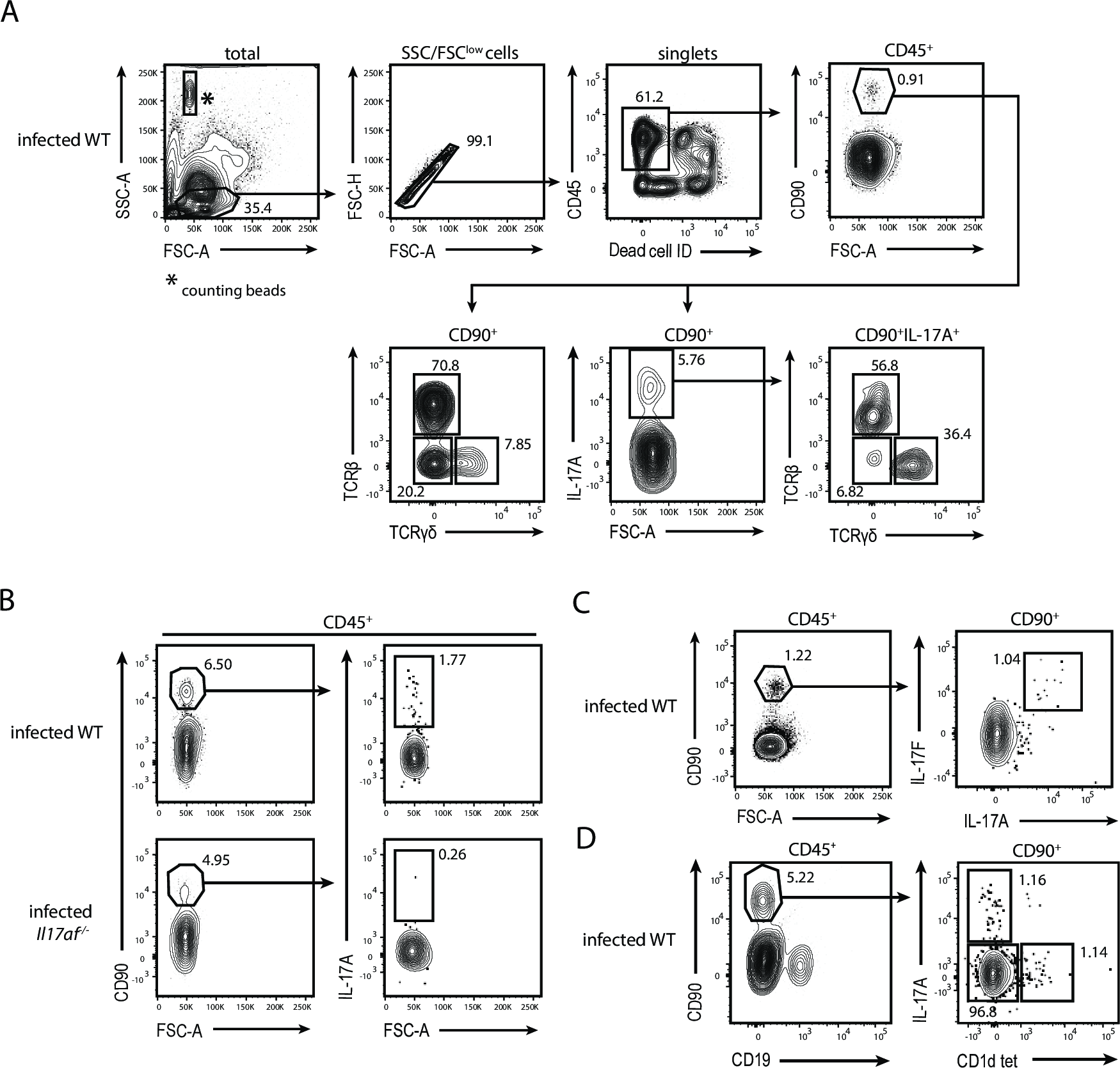

Supplement: S1 Fig — (A) Gating strategy for identifying CD90+IL-17A+ cell populations in the tongue of infected mice. (B) Analysis of CD90+IL-17A+ cells in the tongue of infected WT and Il17af-/- animals. (C) Analysis of IL-17A and IL-17F co-expression by CD90+ cells in infected WT animals. (D) Analysis of IL-17A and CD1d expression by CD90+ cells in infected WT animals. Data shown in B-D are representative of one out of two independent experiments. Pre-gating is on CD45+ cells. Numbers indicate the % of parent in each gate. (TIF) [file ppat.1007069.s001.tif]

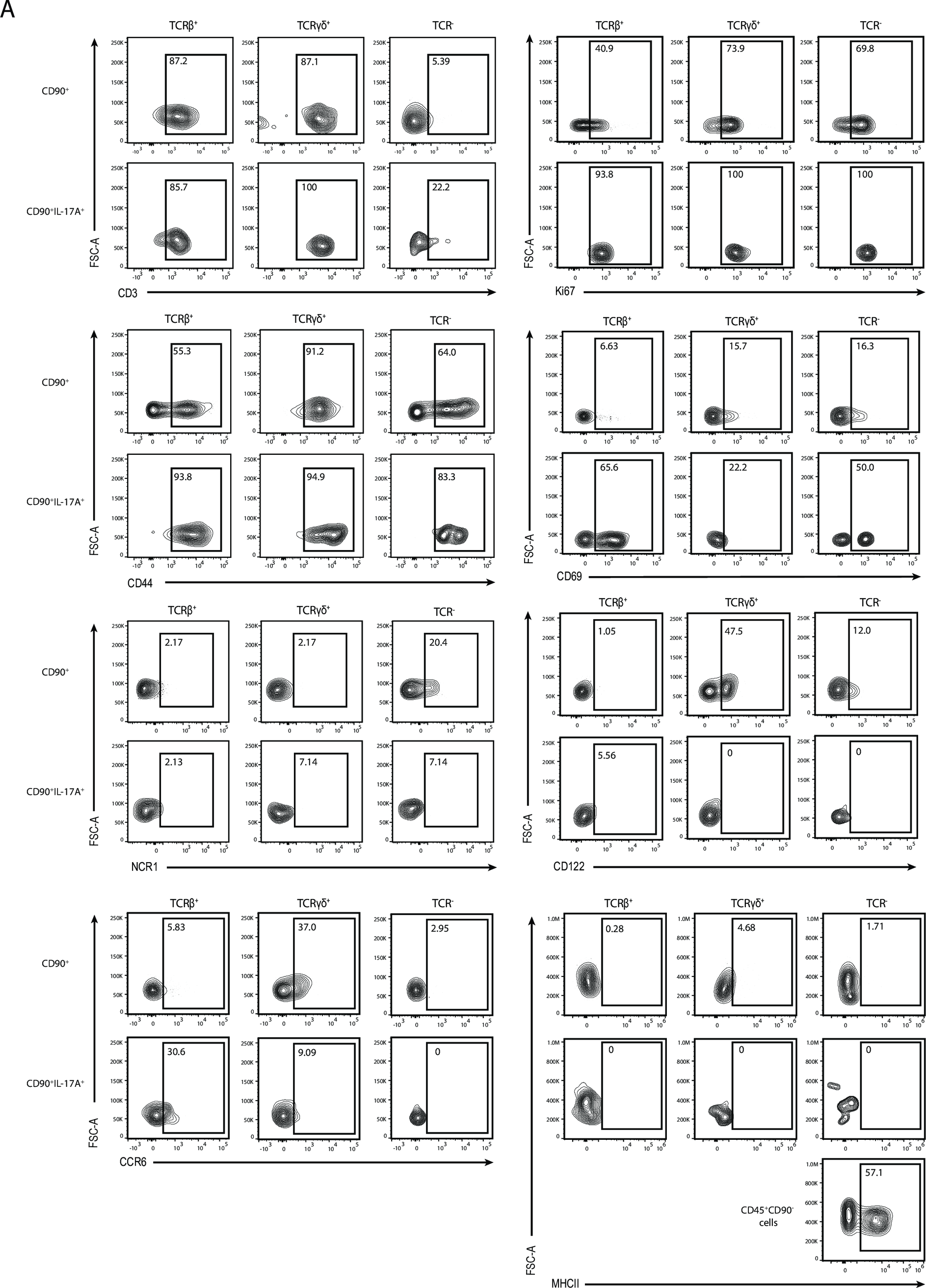

Supplement: S2 Fig — (A) Flow cytometric analysis of the TCRβ+, TCRγδ+ and TCR- subsets for the indicated markers. Pre-gating is on CD45+CD90+ (upper panels) or CD45+CD90+IL-17A+ cells (lower panels). Representative plots from one out of two or three independent experiments are shown. Numbers indicate the % of cells in the gate. (TIF) [file ppat.1007069.s002.tif]

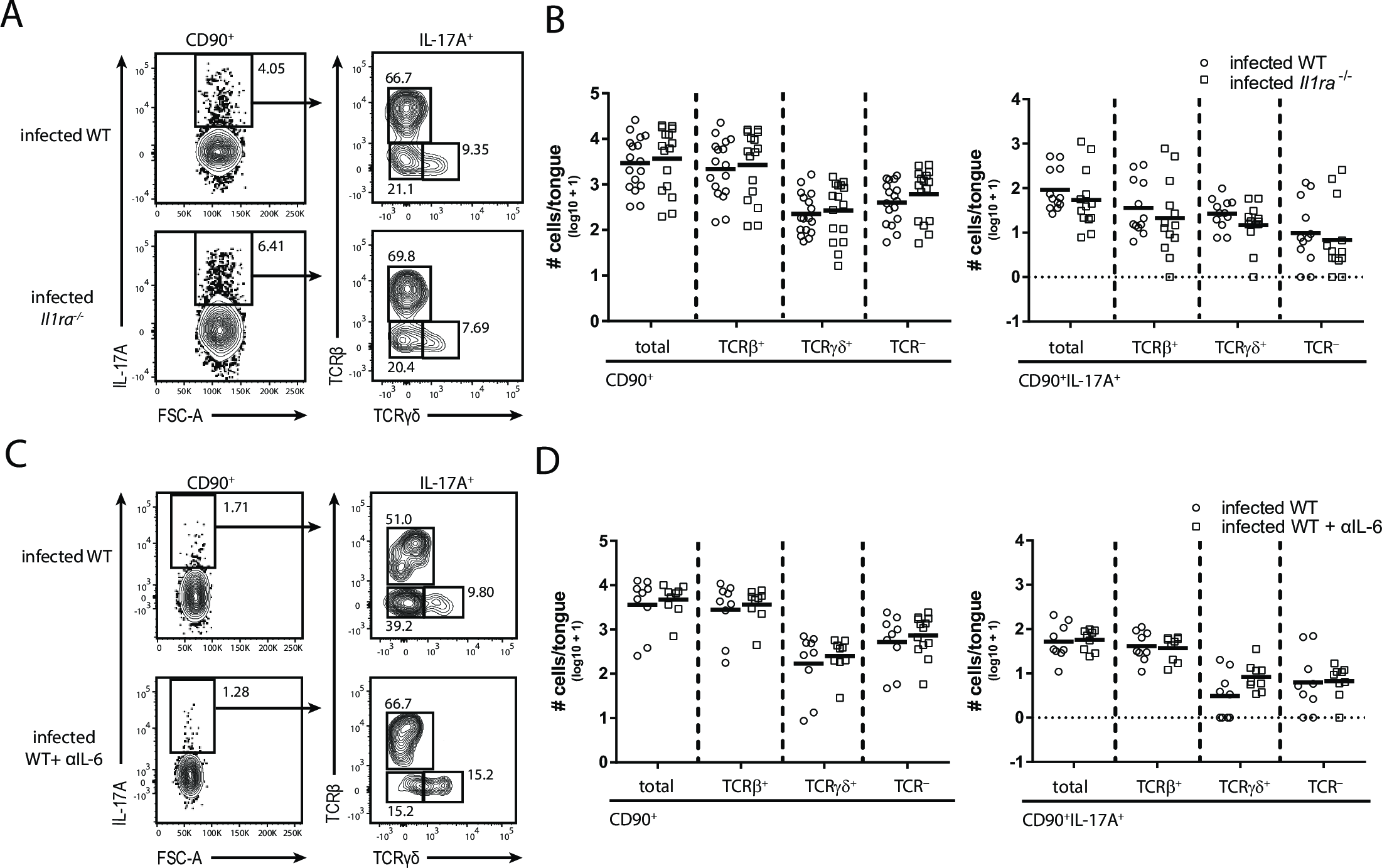

Supplement: S3 Fig — IL-17A+ cellular subsets in the tongue of infected WT and Il1ra-/- animals (A-B) or IL-17A+ cellular subsets in the tongue of infected WT mice that were treated with anti-IL-6 antibody or left untreated (C-D). (A, C) Representative plots. Pre-gating is on CD45+CD90+ cells. Numbers indicate the % of parent in each gate. (B, D) Summary graphs with the total CD90+ and CD90+IL-17A+ cells as well as the respective TCRβ+, TCRγδ+ and TCR- subsets according to the analysis shown in A and C. Each dot represents one animal, the mean of each group is indicated. Graphs show pooled data from three independent experiments. (TIF) [file ppat.1007069.s003.tif]

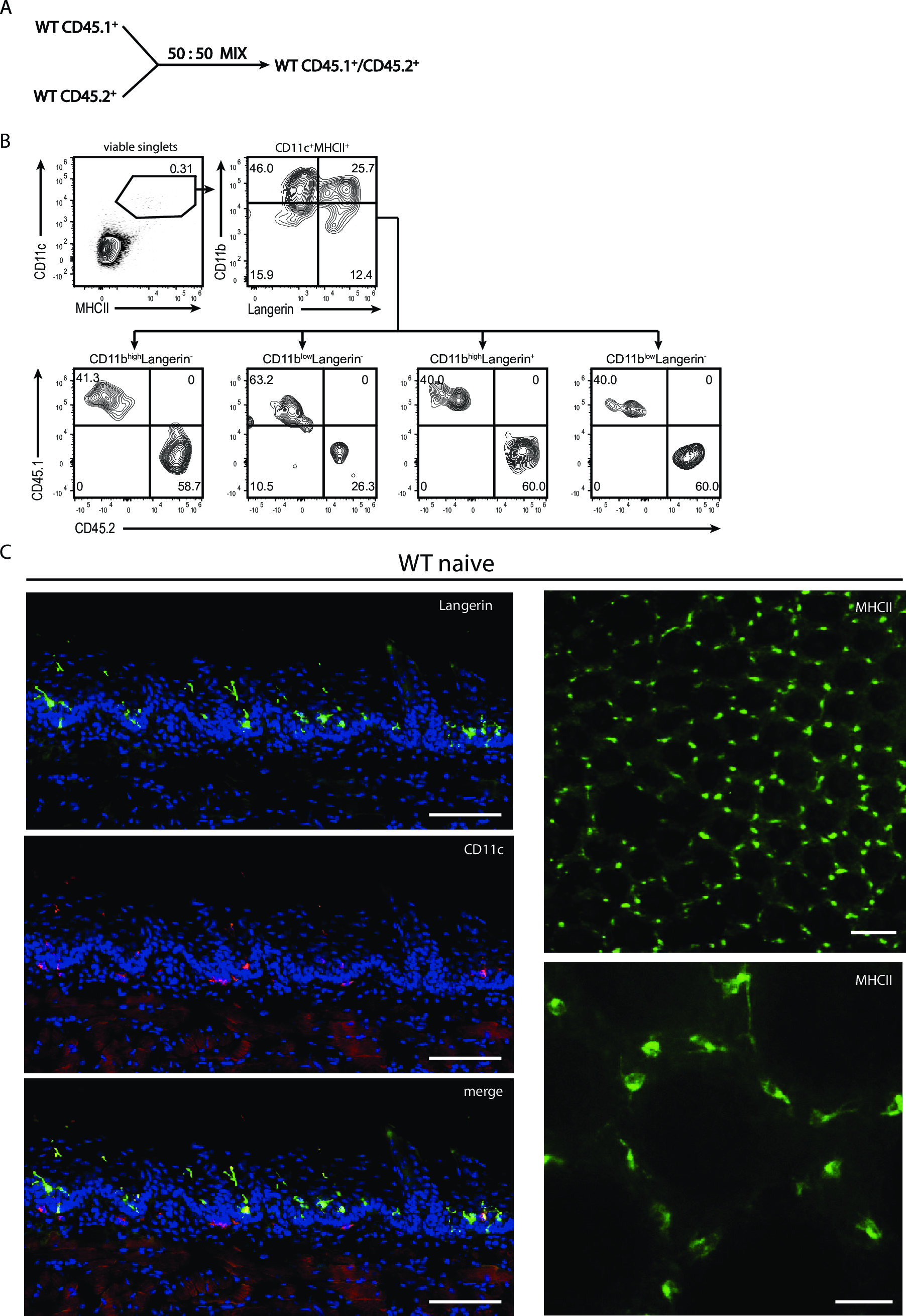

Supplement: S4 Fig — (A) Experimental scheme for bone marrow chimera to investigate radio-resistance of tongue-resident CD11c+MHCII+ MNPs. (B) Flow cytometric analysis of tongue-resident CD11c+MHCII+ MNP subsets for the expression of CD45.1 and CD45.2 in naïve, reconstituted WT animals. Representative plots from one out of two independent experiments are shown. Numbers indicate the % of cells in the gate. (C) Microscopy analysis of MNPs for their expression of Langerin and CD11c in sagittal sections (left, scale bar = 100μm) and for MHCII in epithelial sheets of the tongue from naïve WT mice (right, scale bar = 100μm (top) and 30μm (bottom)). Representative images from one out of two independent experiments are shown. (TIF) [file ppat.1007069.s004.tif]

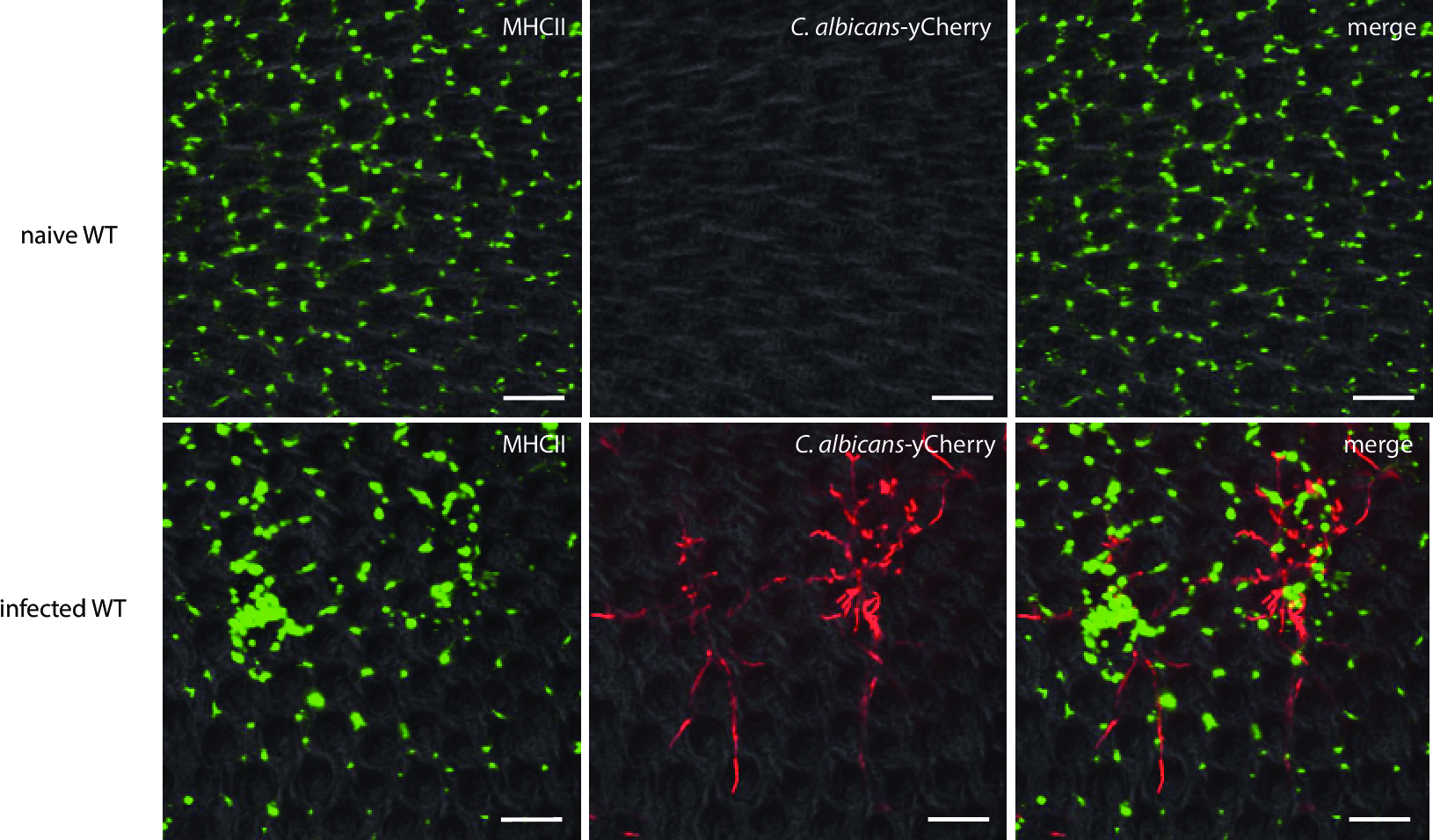

Supplement: S5 Fig — WT mice were infected with yCherry-expressing C. albicans strain CAF-yCherry. Microscopy analysis of C. albicans (red) and MHCII+ MNPs (green) in epithelial sheets obtained from naïve and infected WT mice. Representative pictures from one out of two independent experiments are shown (scale bar = 100μm). (TIF) [file ppat.1007069.s005.tif]

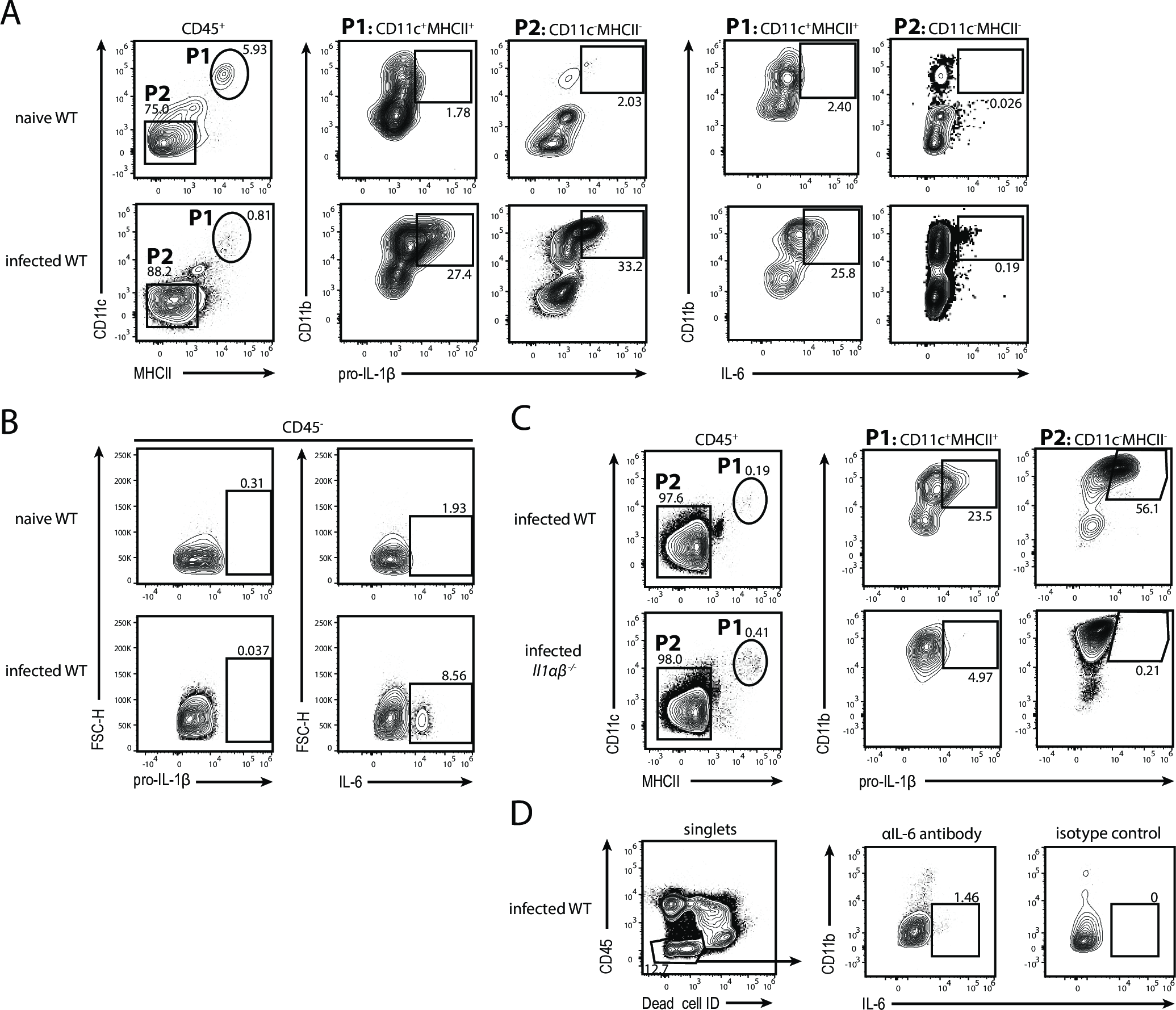

Supplement: S6 Fig — (A) Expression of pro-IL-1β and IL-6 was analyzed by flow cytometry in CD11c+MHCII+ (P1) and CD11c-MHCII- populations (P2) in the tongue of naïve and infected WT mice. Pre-gating is on CD45+ cells. (B) Flow cytometric analysis of CD45- tongue cells from naïve and infected WT mice for pro-IL-1β and IL-6 expression. Pre-gating is on CD45- cells. (C) The specificity of the pro-IL-1β staining was verified by comparing the antibody staining of cells from infected WT with infected Il1ab-/- animals. Pre-gating is on CD45+ cells. (D) The specificity of the IL-6 staining was assessed by means of an isotype control antibody as shown for CD45- cells. Numbers indicate the % of parent in each gate. Representative plots from one out of two independent experiments with 2 animals each are shown in each panel. (TIF) [file ppat.1007069.s006.tif]

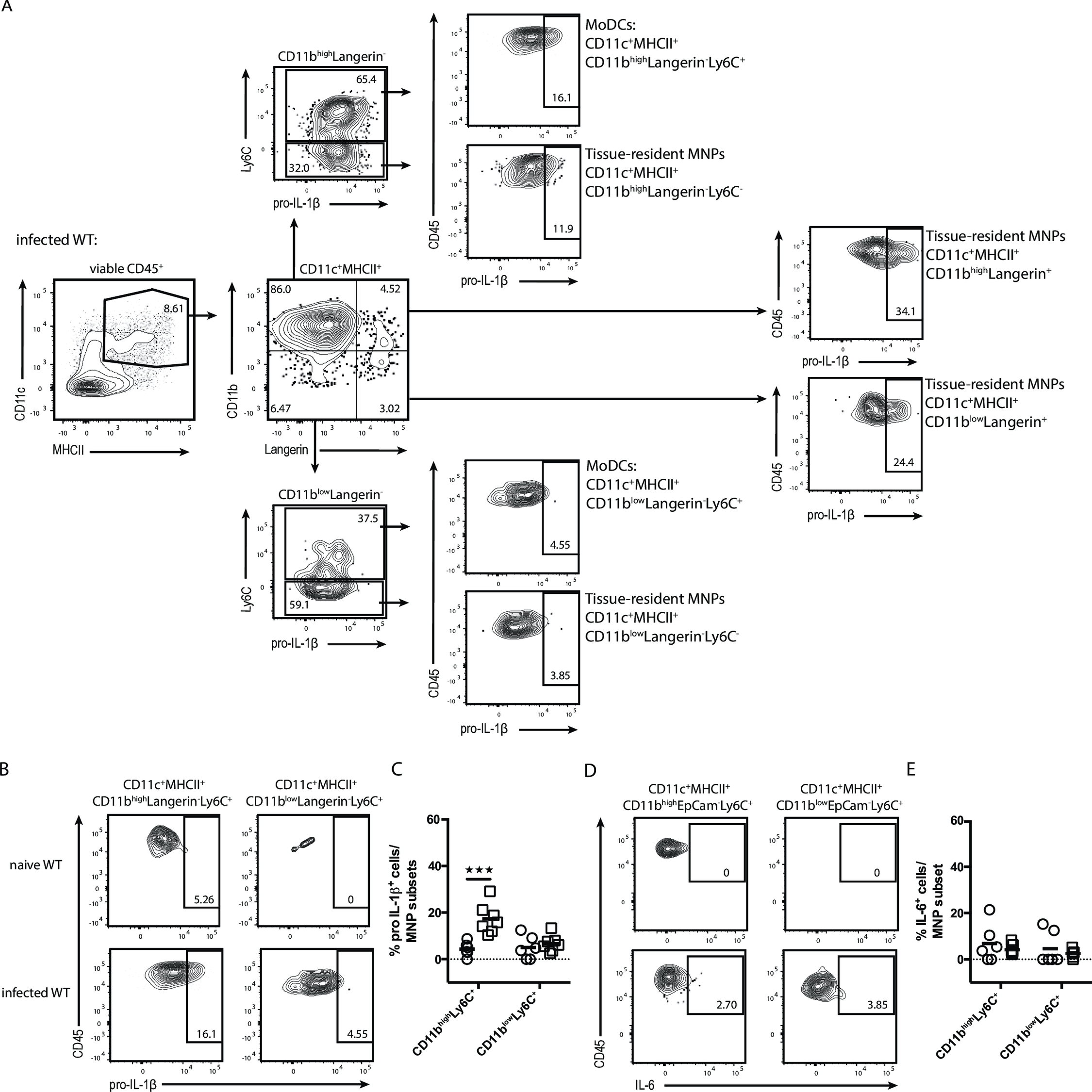

Supplement: S7 Fig — (A) Gating strategy for identifying pro-IL-1β-expressing cells in CD11c+MHCII+ MNP subsets in the tongue of infected WT mice. Cells were gated on viable CD45+ cells. Numbers indicate the % of cells in the gate. The same gating strategy was used to identify IL-6-expressing cells in CD11c+MHCII+ MNP subsets. (B—E) Flow cytometric analysis and summary graphs of pro-IL-1β or IL-6 expression in Ly6C+ inflammatory monocytes in the tongue of naïve and infected WT animals. (B, D) Representative flow cytometric analysis of pro-IL-1β (B) or IL-6 (D) expression in CD11c+MHCII+CD11bhighLy6C+ and CD11c+MHCII+CD11blowLy6C+ inflammatory monocytes in the tongue of naïve and infected WT animals. Representative plots are from one out of two independent experiments. Numbers in the dot plots indicate the % of cells in the gate and are summarized in the graph. (C, E) Summary plots with percentages of cytokine-expressing monocytes in naïve versus infected WT mice. Each symbol represents one animal, the mean of each group is indicated. Data are pooled from two independent experiments. (***p<0.001). (TIF) [file ppat.1007069.s007.tif]

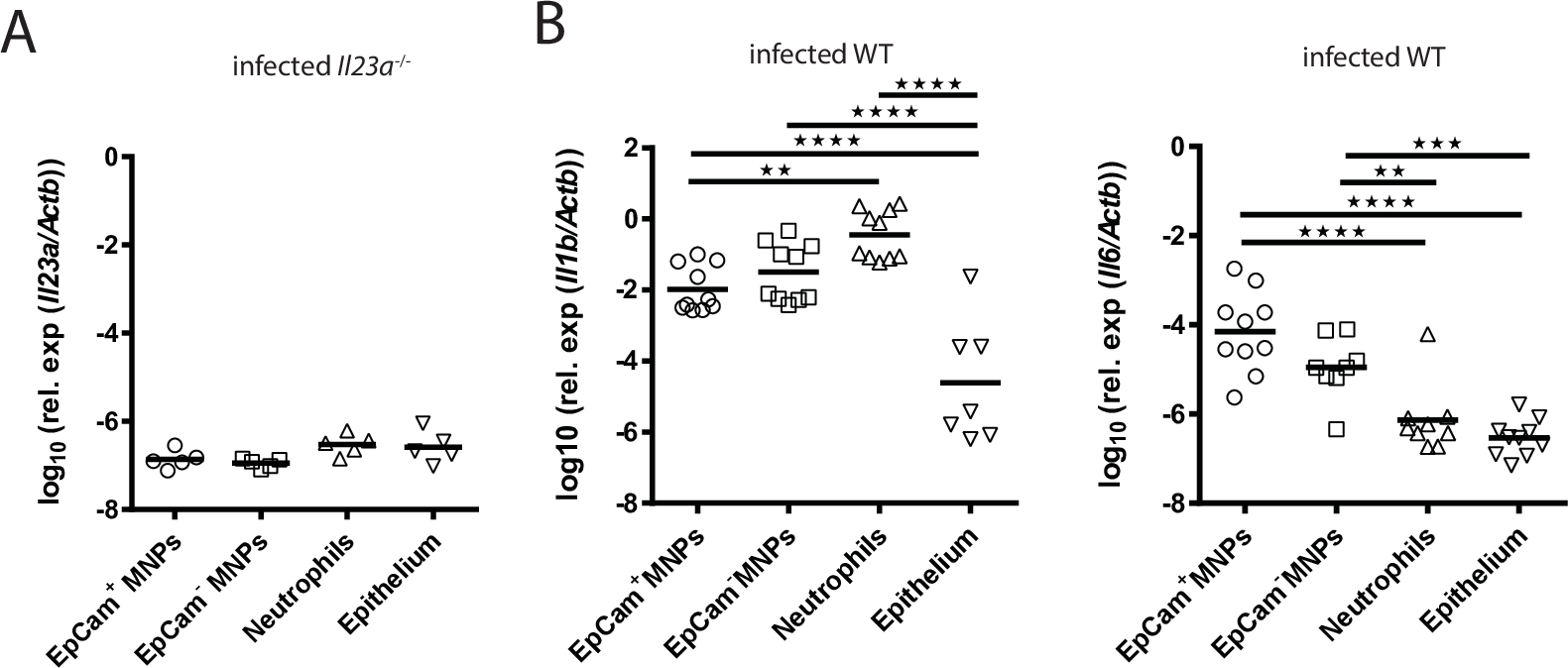

Supplement: S8 Fig — (A, B) RT qPCR analysis of Il23a (A), Il1b (B, left) and Il6 (B, right) expression in sorted CD11c+MHCII+EpCam+ MNPs, CD11c+MHCII+EpCam- MNPs, neutrophils and epithelial cells from infected WT (B) or Il23a-/- mice (A). Each symbol represents one animal, the mean of each group is indicated. Data are pooled from two independent experiments (**p<0.01, ***p<0.001, ****p<0.0001). (TIF) [file ppat.1007069.s008.tif]

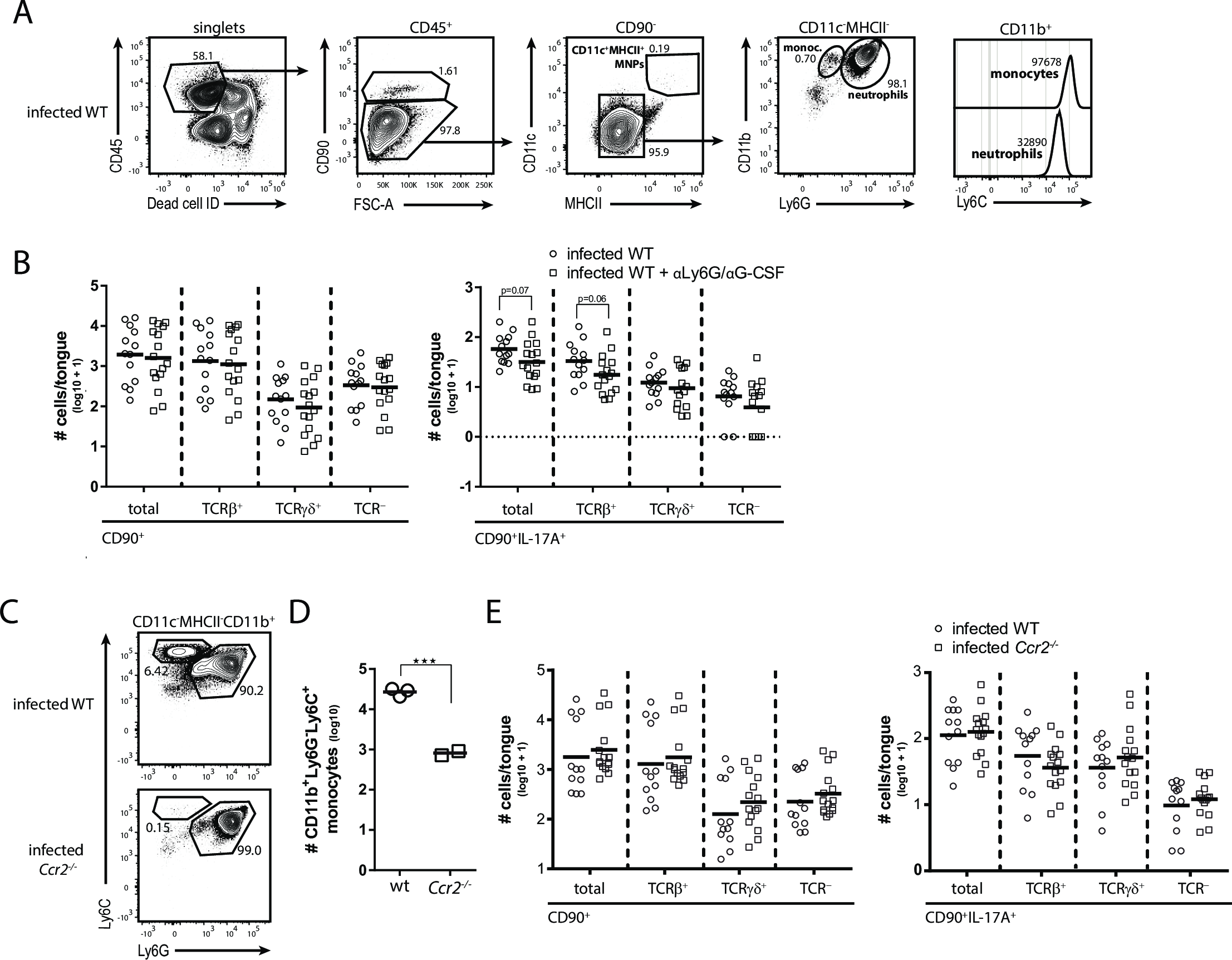

Supplement: S9 Fig — (A) Gating strategy for identifying myeloid cell populations in the tongue of infected mice, including CD90-CD11c+MHCII+ MNPs, CD11c-MHCII-CD11b+Ly6G+ neutrophils and CD11c-MHCII-CD11b+Ly6Chigh monocytes. Numbers indicate the % of cells in the gate. Numbers in the histograms in A indicate MFI (median) of Ly6C. (B) Summary plot with absolute numbers of CD90+ and CD90+IL-17A+ cells and the respective TCRβ+, TCRγδ+ and TCR- subsets isolated from the tongue of infected WT mice that were or were not treated with anti-G-CSF and anti-Ly6G antibodies. Each dot represents one animal. The mean of each group is indicated. Graphs show pooled data from three independent experiments. (C—E) Flow cytometric analysis of Ly6C+Ly6G- monocytes in the tongue of infected WT and Ccr2-/- mice. Pre-gating is on CD45+CD90-CD11c-MHCII-CD11b+ cells. Representative FACS plots are shown in C and summary graph with absolute numbers of CD11b+Ly6C+Ly6G- monocytes is shown in D. Each symbol represents one animal, the mean of each group is indicated. Data are from one out of two independent experiments. (E) Absolute numbers of CD90+ and CD90+IL-17A+ cells and the respective TCRβ+, TCRγδ+ and TCR- subsets in the tongue of infected WT or Ccr2-/- mice. Each symbol represents one animal, the mean of each group is indicated. Graphs show pooled data form three independent experiments. (***p<0.001). (TIF) [file ppat.1007069.s009.tif]
